# DTT-Induced Transcriptome Profiling Reveals Divergent ER Proteostasis and Stress-Adaptation Programs in CHO-S/HB8 and CHO-4BGD/DUL Producer Clones

**DOI:** 10.3390/ijms27167427

**Published:** 2026-08-19

**Authors:** Nadezhda A. Orlova, Nadezhda A. Potapova, Rolan R. Shaifutdinov, Ivan I. Vorobiev

**Affiliations:** 1 Laboratory of Mammalian Cell Bioengineering, Skryabin Institute of Bioengineering, Federal Research Centre ‘Fundamentals of Biotechnology’ of the Russian Academy of Sciences, 60 let Oktjabrja pr-t 7, bld. 1, 117312 Moscow, Russia; urtmyakowski@yandex.ru (R.R.S.); ptichman@gmail.com (I.I.V.); 2 Association “Technological Platform BioTech 2030”, Leninsky Prospekt, 33, Bld. 3, 119071 Moscow, Russia

**Keywords:** CHO cells, RNA-seq, DTT, endoplasmic reticulum stress, unfolded protein response, PERK, ISR, XBP1 splicing, ERAD, apoptosis-resistant CHO, secretory pathway, BiP/GRP78

## Abstract

Chinese hamster ovary (CHO) producer cells differ in their capacity to accommodate secretory and endoplasmic reticulum (ER) stress, but how producer and host-cell backgrounds are associated with ER-proteostasis responses remains poorly understood. We compared 24 h dithiothreitol (DTT)-induced reductive ER stress responses in two clonal producers: CHO-S-derived HB8 secreting human chorionic gonadotropin and apoptosis-resistant CHO-4BGD-derived DUL secreting a GLP-1–Fc fusion protein with similar specific productivities. Responses were analyzed by strand-specific RNA-seq, Xbp1-splicing RT-PCR, immunoblotting, functional enrichment, and curated-module analysis. Analysis of the complete three-replicate dataset identified 299 DTT-responsive genes in HB8 and 877 in DUL, demonstrating a broader transcriptional response in the DUL clone. Analysis after exclusion of the atypical HB8#3 matched pair yielded 404 and 1019 differentially expressed genes, respectively, and was used for detailed sensitivity analysis. Both producers showed ISR/ATF4/CHOP-associated transcriptional activation and Xbp1 mRNA splicing, whereas changes in total Xbp1 and Hspa5/BiP transcript abundance were limited. BiP and CHOP accumulation was detected at the protein level, while phospho-eIF2α and ATF6 responses were variable. Baseline RNA-seq data comparison revealed extensive transcriptional divergence between HB8 and DUL. HB8 showed higher expression of several classical ER folding/redox factors, whereas DUL showed higher expression of selected ISR-, quality-control- and stress-survival-associated genes. DUL additionally displayed broader vesicle/endocytic and amino-acid/glutathione-related remodeling. Thus, the producers occupy distinct ER-proteostasis states, and DUL mounts a broader, but not uniformly stronger, canonical UPR response to reductive ER stress.

## 1. Introduction

Chinese hamster ovary (CHO) cells are the dominant mammalian host for production of therapeutic glycoproteins. They combine robust suspension growth with the ability to perform complex protein folding, assembly and post-translational modification. Yet high-level recombinant protein expression places substantial demands on the secretory pathway. Newly synthesized secretory proteins must be co-translationally targeted to the endoplasmic reticulum (ER), translocated through the Sec61 translocon, folded and assembled with the assistance of ER chaperones and protein disulfide isomerases, subjected to glycoprotein quality control, and transported through the ER–Golgi pathway before secretion. Bottlenecks at any of these steps can limit product yield and quality [1,2].

The secretory capacity of CHO cells is therefore not determined only by transgene copy number or mRNA abundance. It also depends on the functional state of ER protein folding, oxidative folding, ER-associated degradation (ERAD), vesicular transport, and stress-response pathways. Comparative transcriptomic and proteomic studies have shown that differences in recombinant protein secretion can be associated with altered expression of genes involved in ER translocation, protein folding, unfolded protein response (UPR) activation, Golgi processing and trafficking, and degradation of aberrant protein species [2,3,4].

When the folding load exceeds ER capacity, cells activate the unfolded protein response (UPR), mediated by the PERK/EIF2AK3, IRE1/ERN1, and ATF6 branches [5,6,7]. This response can be adaptive by reducing translational influx into the ER and increasing folding, degradation, and trafficking capacity. However, prolonged or excessive ER stress can shift the response toward translational repression, apoptosis, autophagy-related remodeling, and loss of culture performance [8,9].

In recombinant CHO cultures, ER stress is closely linked to redox homeostasis, mitochondrial function and protein quality control. This coupling is particularly important for antibodies, Fc-fusion proteins and bispecific or multispecific formats, whose assembly depends on efficient disulfide-bond formation. Sinharoy et al. showed that fed-batch production of a bispecific antibody was associated with mitochondrial dysfunction, glutathione oxidation, stronger ER-stress markers and increased product aggregation compared with perfusion culture [10]. Complementary multi-omics analysis demonstrated that cysteine feed level affects sulfur-amino-acid and glutathione metabolism, cell viability and monoclonal-antibody production in CHO fed-batch cultures [11]. In a separate study, cystine supplementation promoted ER stress, ER-associated degradation and apoptosis, whereas concomitant tyrosine supplementation mitigated these effects and improved culture performance [12]. These findings connect process-associated redox imbalance with ER proteostasis and product quality and provide a rationale for probing redox-sensitive folding capacity in different CHO producer backgrounds.

UPR activation is not necessarily a sign of culture failure. Productive cells may instead engage a controlled adaptive response to secretory load [13]. For example, RNA-seq analysis of an IgG-producing CHO fed-batch culture revealed early induction of UPR-associated genes including HSPA5/BiP, ATF4, DDIT3/CHOP and PPP1R15A/GADD34 [14]. Similarly, engineering approaches targeting secretory pathway components, including SRP-dependent translocation and XBP1/ERO1-mediated expansion of ER folding capacity, have been used to improve secretion of difficult-to-express proteins [15,16].

Recent advances in genome-engineered CHO host cells have shown that long-term culture performance can also be improved by targeted modulation of apoptosis and autophagy pathways. CHO 4BGD cells were generated from CHO-S by CRISPR/Cas-mediated disruption of pro-apoptotic genes Bax and Bak1, together with disruption of DHFR and GLUL and overexpression of BCL2 and BECN1. This engineered background shows extended culture longevity, increased integral cell density and altered stress-response dynamics during prolonged cultivation [17]. This background therefore provides a useful system for testing whether disruption of BAX/BAK1-dependent intrinsic apoptosis alters transcriptional adaptation to secretory stress.

In this study, we compared two recombinant CHO producers derived from distinct host backgrounds: HB8 from CHO-S and DUL from the apoptosis-resistant CHO 4BGD line [18]. We hypothesized that disruption of BAX/BAK1-dependent intrinsic apoptosis in the 4BGD background would alter cellular adaptation to ER stress. We used RNA-seq to compare the basal organization of secretory and stress-response pathways and their remodeling after DTT-induced reductive stress. DTT perturbs thiol-disulfide homeostasis and directly challenges oxidative protein folding in the ER [19]. This mechanism is relevant to both disulfide-containing products examined here. We therefore tested whether the two producer lines differed in their activation of UPR, ER quality-control, redox and broader stress-adaptation programs under the same folding challenge.

## 2. Results

### 2.1. RNA-Seq Analysis Reveals a Broader DTT-Induced Transcriptional Response in DUL than in HB8

**Figure 1:**
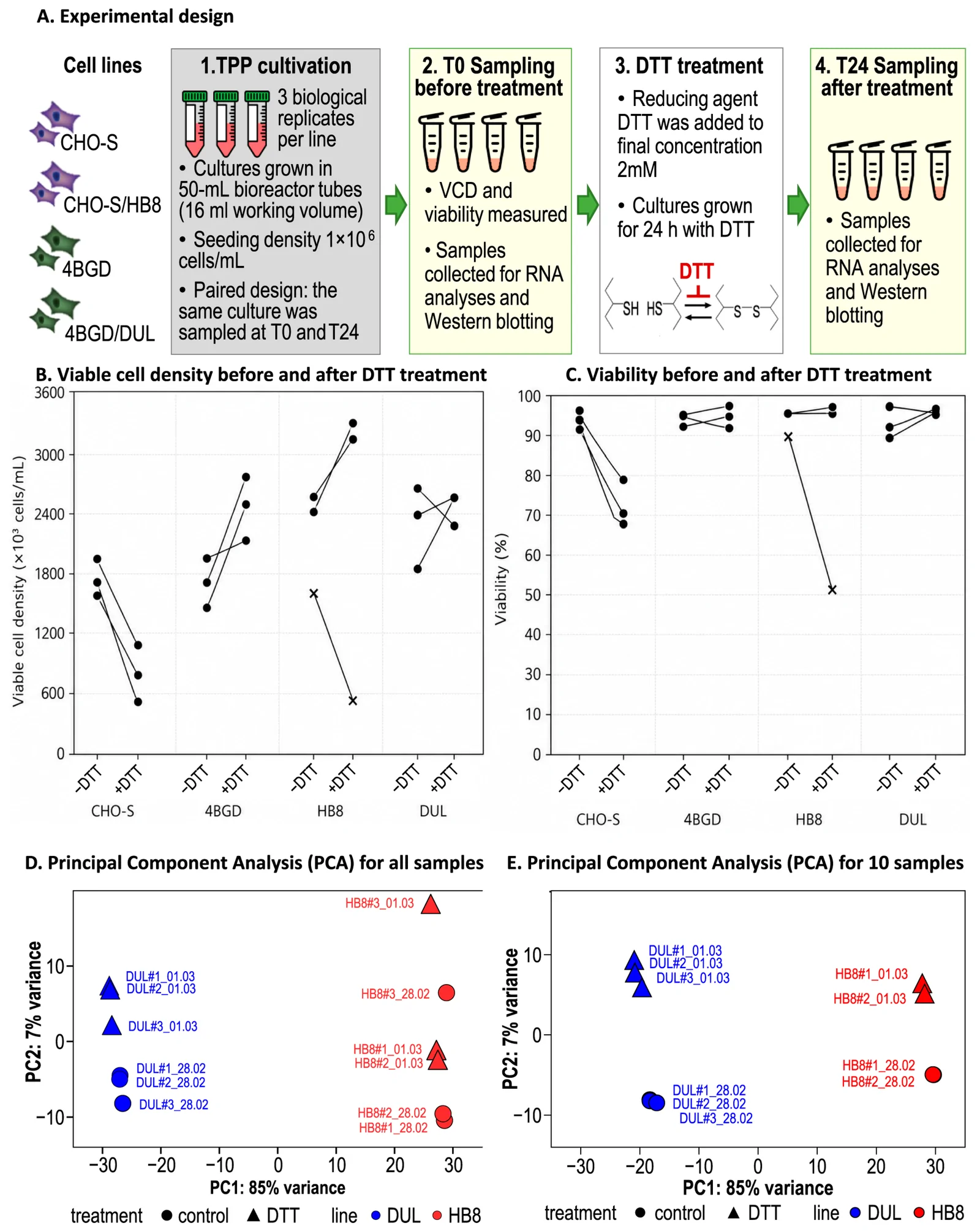
Experimental design and RNA-seq sample quality assessment. (**A**) Paired baseline/post-DTT sampling design. Four cell lines were analyzed by validation assays, whereas RNA-seq was performed for HB8 and DUL. (**B**,**C**) Viable cell density and viability at baseline and after 24 h of DTT exposure; lines connect matched cultures. The excluded HB8 replicate pair is indicated. (**D**) Principal component analysis (PCA) of all RNA-seq libraries, showing separation by producer line and displacement of HB8 replicate pair 3 along PC2. (**E**) PCA after exclusion of HB8 replicate pair 3. Underlying per-replicate viable cell density and viability values are provided in Supplementary Dataset S1.

To compare transcriptional responses to reductive ER stress in distinct CHO producer backgrounds, we performed RNA-seq of the CHO-S-derived HB8 and apoptosis-resistant CHO 4BGD-derived DUL cell lines at baseline and after 24 h of DTT exposure. The paired sampling design and culture characteristics at both time points are summarized in Figure 1A–C.

To independently evaluate whether the applied DTT treatment caused extensive membrane damage, the experimental conditions were reproduced in a separate follow-up experiment using newly cultured HB8 and DUL cells. These cultures were not the same samples as those used for RNA-seq, Western blotting or Figure 1. The calculated cytotoxicity index was −3.82 ± 1.97% for HB8 and 0.19 ± 0.55% for DUL. Negative values indicate that extracellular LDH activity after DTT treatment did not exceed the spontaneous LDH release measured in the corresponding untreated culture. Thus, the cytotoxicity index remained close to zero in both producer lines, providing no evidence of DTT-induced cytolytic membrane damage under the applied conditions (Figure S2).

Principal component analysis (PCA) identified distinct expression profiles for both samples of HB8 replicate pair 3, which were displaced concordantly from the remaining HB8 samples along PC2 (Figure 1D). The corresponding culture also showed lower viable cell density and viability than the other HB8 replicates both before and after DTT treatment (Figure 1B,C; Supplementary Dataset S1), indicating an atypical culture state. The two HB8#3 libraries had the highest mean Cook’s distances among all samples, with values of 0.132 and 0.164 compared with a maximum of 0.086 among the remaining libraries (Supplementary Table S3). In contrast, sequencing quality, read retention, mapping efficiency and gene-assignment rates remained within the ranges observed for the other libraries (Supplementary Table S2). PCA performed after exclusion of HB8#3 retained the separation of the experimental groups (Figure 1E). The complete three-replicate dataset was retained for the primary comparison of response magnitude. The analysis was additionally repeated after exclusion of HB8#3 to examine the influence of this biologically atypical matched pair on detailed differential-expression patterns.

Because removal of a matched replicate pair also reduces the sample size and changes the dataset used for normalization and dispersion estimation, we examined whether a comparable effect would be observed after alternatively excluding the non-outlying DUL#3 pair. In the complete 3-versus-3 dataset, 299, 877, 1607 and 1789 significant genes were detected in the HB8 DTT versus control, DUL DTT versus control, HB8 versus DUL under control conditions and HB8 versus DUL under DTT conditions, respectively. Exclusion of HB8#3 increased these counts to 404, 1019, 1879 and 2106, whereas exclusion of DUL#3 reduced them to 242, 599, 1387 and 1561, respectively. Thus, the complete dataset retained the principal qualitative finding of a broader DTT-induced transcriptional response in DUL than in HB8. Because exact threshold-based DEG counts were sensitive to sample composition, the HB8#3-excluded analysis was subsequently used as a secondary sensitivity analysis for detailed gene- and pathway-level exploration. The increased detection of differential expression after removal of HB8#3 was not reproduced by reducing the dataset through exclusion of a non-outlying matched pair. At the same time, the magnitude of the changes observed after either exclusion illustrates the sensitivity of threshold-based DEG counts to sample composition when only two or three biological replicates are available. We therefore used this analysis to assess the robustness of the overall response patterns rather than treating the exact numbers of significant genes as independent evidence for sample exclusion.

**Figure 2:**
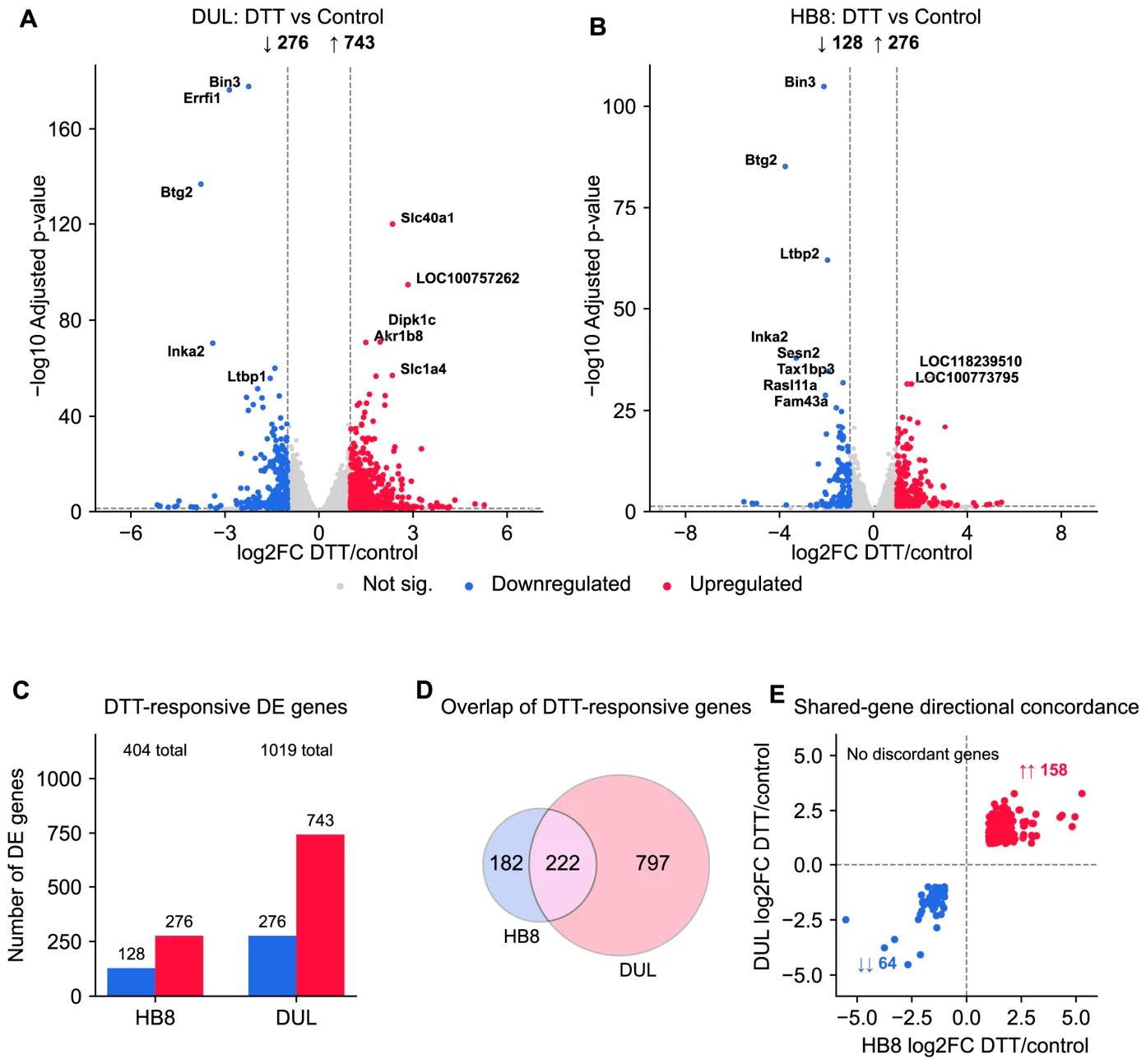
Global transcriptional response to DTT in HB8 and DUL producers. (**A**,**B**) Volcano plots showing differential expression after DTT treatment in DUL and HB8. Red points indicate genes significantly upregulated after DTT, blue points indicate genes significantly downregulated after DTT, and grey points indicate genes not passing the significance threshold. Dashed vertical lines indicate log2 fold change thresholds of −1 and +1; the horizontal dashed line indicates adjusted *p*-value = 0.05; selected genes are labeled. Positive log2FC values indicate induction by DTT, whereas negative values indicate repression. (**C**) Number of significantly differentially expressed genes after DTT treatment in HB8 and DUL producers. Genes were considered significant at an adjusted *p*-value < 0.05 and an absolute log2 fold change > 1. Red bars indicate genes upregulated after DTT treatment, and blue bars indicate genes downregulated after DTT treatment. (**D**) Venn diagram showing overlap of significant DTT-responsive gene sets. (**E**) Directional concordance of the 222 shared DTT-responsive genes. Each point represents one shared gene, plotted by its log2 fold change in HB8 and DUL. Differential expression was defined as adjusted *p* < 0.05 and |log2FC| > 1. The differential-expression results shown in this figure were obtained from the HB8#3-excluded sensitivity dataset (2 × 3). Upward and downward arrows in all figures indicate the numbers of significantly upregulated and downregulated genes, respectively.

In the HB8#3-excluded sensitivity analysis, differential expression analysis was performed using DESeq2. Genes were considered significantly differentially expressed at an adjusted *p*-value < 0.05 and an absolute log2 fold change > 1. DTT treatment induced 404 differentially expressed genes in HB8, including 276 genes upregulated and 128 genes downregulated after treatment. In DUL, the DTT-induced transcriptional response was substantially broader and included 1019 differentially expressed genes, of which 743 were upregulated and 276 were downregulated after treatment. (Table 1, Figure 2A–C) Thus, the number of DTT-responsive genes in DUL was approximately 2.5-fold higher than in HB8, indicating that the two producer lines differed substantially in the extent of transcriptional remodeling during acute ER redox stress. Complete gene-level differential-expression results for the complete dataset and both sensitivity analyses are provided in Supplementary Dataset S2.

Comparison of the two DTT-responsive gene sets revealed a shared core of 222 genes that were significantly regulated in both producers. These common genes represented more than half of the HB8 response but only a smaller fraction of the DUL response, indicating that most DUL-regulated genes belonged to an additional line-specific program. Specifically, 182 genes were significant only in HB8, whereas 797 genes passed the significance and effect-size thresholds only in DUL (Figure 2D). Importantly, all 222 shared DTT-responsive genes changed in the same direction in both cell lines (Figure 2E). Among them, 158 genes were upregulated in both HB8 and DUL, whereas 64 genes were downregulated in both lines, and no discordant genes were detected. This complete directional concordance indicates that HB8 and DUL activate a common core DTT-response program, but that DUL additionally engages a much broader transcriptional response.

The shared response included stress-associated genes linked to the integrated stress response and adaptive redox remodeling, including *Trib3*, *Chac1*, *Slc7a11*, *Sesn2* and *Ddit4*. Several additional UPR-related transcripts, including *Atf4* and *Ddit3/Chop*, were also significantly regulated at the adjusted *p*-value level, although not all of them passed the two-fold effect-size threshold in both cell lines. Thus, the common DTT response comprised a concordant stress-associated core, whereas the markedly larger DUL-specific component indicated broader transcriptional remodeling in the apoptosis-resistant producer. These genes should be regarded as candidates rather than established mediators of stress tolerance. SLC7A11 can support cystine uptake and resistance to oxidative stress [20], whereas CHAC1 degrades glutathione [21], and TRIB3 has been implicated in ER-stress-induced cell death [22]. Their concurrent induction may therefore represent interacting adaptive and stress-promoting components of the response. Establishing their roles in the stress responses of HB8 and DUL cells will require individual and combinatorial KO or overexpression followed by measurements of survival, recombinant-protein secretion and specific productivity under stress.

Together, these results show that HB8 and DUL share a highly concordant core response to DTT but differ markedly in the scale of stress-induced transcriptional remodeling. The CHO-S-derived HB8 producer displayed a narrower response, whereas the 4BGD-derived DUL producer activated a substantially larger additional gene set. These global differences motivated subsequent analysis of UPR branch activation and of the broader functional programs underlying the two producer-specific responses.

### 2.2. DTT Activates UPR Branches at Distinct Regulatory Levels

#### 2.2.1. RNA-Seq Indicates Integrated Stress Response (ISR)/ATF4/CHOP-Associated Transcriptional Activation but Weak BiP-Centered Chaperone Induction

**Figure 3:**
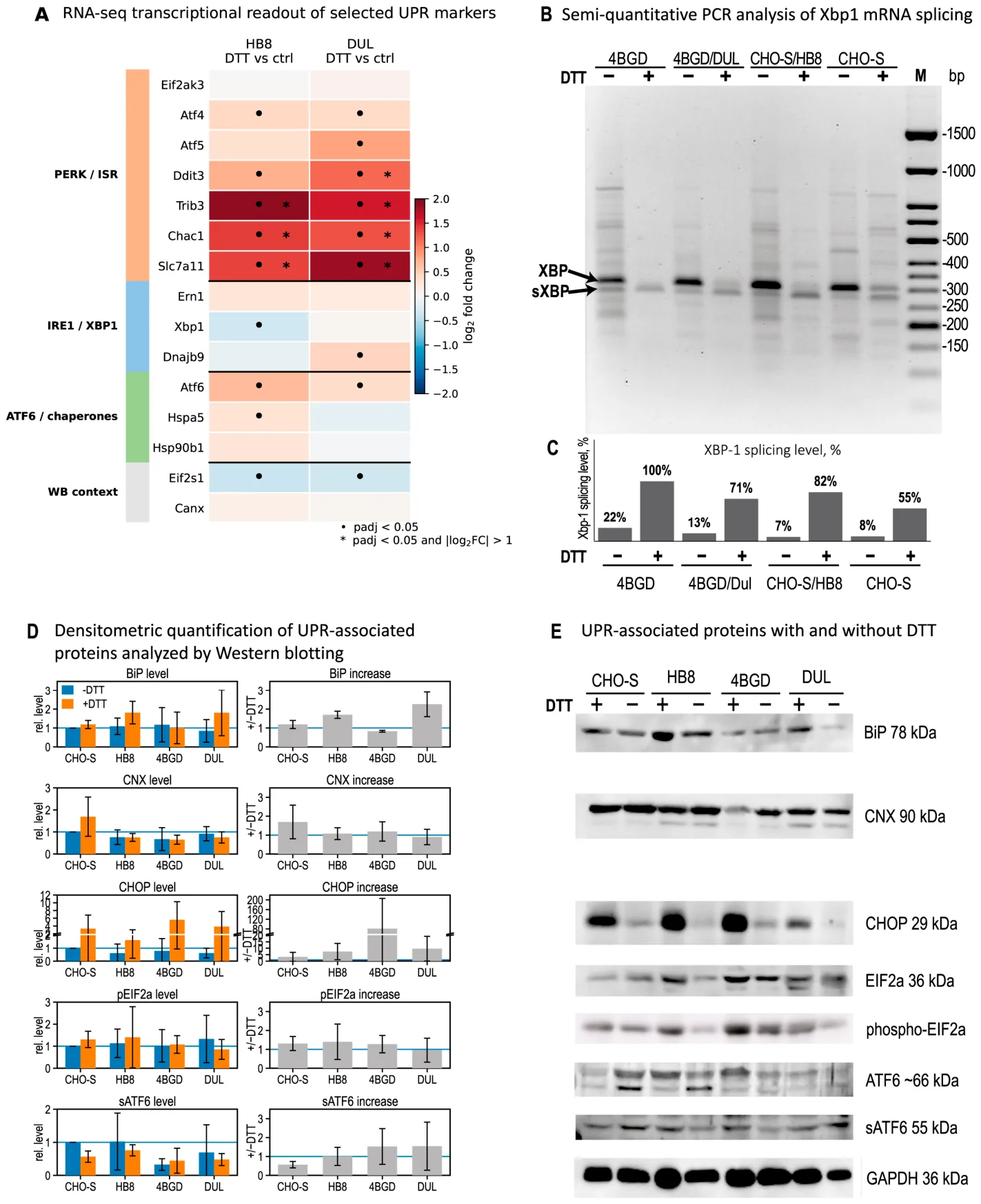
UPR branch activation at transcriptional and post-transcriptional levels after DTT treatment. (**A**) DESeq2 log2FC values for DTT-treated versus untreated cells for selected genes representing the PERK/ISR, IRE1/XBP1 and ATF6/chaperone branches, together with *Eif2s1* and *Canx* included as transcript-level context for proteins analyzed by Western blotting. Dots indicate adjusted *p* < 0.05; asterisks additionally indicate |log2FC| > 1. (**B**) Representative semi-quantitative RT-PCR analysis of *Xbp1* mRNA splicing in parental and producer lines under control and DTT conditions. Bands corresponding to unspliced *Xbp1* (*Xbp1u*, 305 bp) and spliced *Xbp1* (*Xbp1s*, 279 bp) are indicated. M, molecular size marker. (**C**) Densitometric XBP1-splicing fraction calculated as Xbp1s/(Xbp1s + Xbp1u) × 100% for the gel in B. (**D**) Densitometric quantification of immunoblots; data are shown as means ± SD. Levels were normalized to the untreated CHO-S sample, and DTT-induced changes were calculated from GAPDH-normalized band intensities. Three immunoblots for each antigen were obtained from independently cultured and treated cell samples. Quantification is presented descriptively; no inferential statistical comparisons were performed. (**E**) Representative immunoblots for BiP/GRP78, calnexin, CHOP/DDIT3, total eIF2α, phospho-eIF2α, cleaved ATF6 and full-length ATF6 in parental and producer lines under control and DTT conditions; GAPDH is the loading control. RNA-seq log2FC values in panel (**A**) were obtained from the HB8#3-excluded sensitivity dataset. Original Western blot membrane images and raw densitometry data are presented in Supplementary Materials File S1.

RNA-seq analysis revealed a branch-selective UPR-associated transcriptional response to DTT in both producer cell lines (Figure 3A). The clearest response involved the ISR/ATF4/CHOP-associated module. *Atf4* transcript abundance increased significantly in both HB8 and DUL, although the magnitude of change was below the two-fold threshold used for global DE gene classification (log2FC = 0.42, adjusted *p* = 0.0024 in HB8; log2FC = 0.39, adjusted *p* = 1.5 × 10^−4^ in DUL). *Ddit3/Chop* was also induced in both producers and exceeded the two-fold threshold in DUL (log2FC = 0.73 in HB8 and 1.15 in DUL), whereas *Trib3* showed strong induction in both lines (log2FC = 1.89 in HB8 and 1.56 in DUL). *Atf5* displayed a more DUL-biased response, with significant induction in DUL (log2FC = 0.81, adjusted *p* = 4.0 × 10^−12^) but only a weaker, non-significant increase in HB8. In contrast, *Eif2ak3/Perk* itself was not transcriptionally induced by DTT in either producer, although its basal expression was substantially higher in DUL than in HB8. The ATF6/chaperone-associated transcript response was more limited. *Atf6* increased significantly in both producers, with a larger change in HB8 (log2FC = 0.65) than in DUL (log2FC = 0.35). *Hspa5/BiP* showed only a small increase in HB8 (log2FC = 0.28) and no induction in DUL. *Hsp90b1/Grp94* did not change significantly in either line. Total *Xbp1* transcript abundance likewise did not increase after DTT; it decreased modestly in HB8 (log2FC = −0.33, adjusted *p* = 0.0045) and remained unchanged in DUL. Thus, at the transcript level, the most prominent UPR-associated signature was an ISR/ATF4/CHOP-related response rather than broad induction of a canonical BiP-centered ER chaperone program (Figure 3A).

#### 2.2.2. XBP1 Splicing Confirms IRE1/XBP1 Activation Despite Weak Changes in Total Xbp1 Transcript Abundance

Because total *Xbp1* RNA-seq counts did not indicate activation of the IRE1/XBP1 branch, *Xbp1* mRNA processing was examined directly by semi-quantitative RT-PCR (Figure 3B,C). The assay resolved the unspliced 305-bp *Xbp1u* product and the shorter 279-bp *Xbp1s* product, and the spliced fraction was quantified relative to total amplified *Xbp1* product as *Xbp1s*/(*Xbp1s* + *Xbp1u*), consistent with the general normalization logic used in previous gel-based XBP1-splicing analyses [23]. Basal XBP1 splicing was low in all four cell lines but differed between backgrounds, reaching 22% in 4BGD, 13% in 4BGD/DUL, 7% in CHO-S/HB8 and 8% in CHO-S. DTT markedly increased the spliced fraction in every line, to 100% in 4BGD, 71% in 4BGD/DUL, 82% in CHO-S/HB8 and 55% in CHO-S (Figure 3B,C). These changes corresponded to increases of 78, 58, 75 and 47 percentage points, respectively. Thus, direct analysis of *Xbp1* processing demonstrated robust IRE1/XBP1 activation in both CHO-S- and 4BGD-derived backgrounds despite the absence of increased total *Xbp1* transcript abundance in the RNA-seq data. Because the values in Figure 3C were obtained by densitometry of the representative gel shown in Figure 3B, they are presented descriptively without inferential statistical analysis.

#### 2.2.3. Western Blotting Reveals Protein-Level UPR Responses That Differ Between Producer and Parental Cell Lines

Western blot quantification across three blots per antigen showed that several UPR-associated responses were detectable at the protein or phosphorylation level and were not directly predicted by steady-state transcript abundance (Figure 3D,E). After normalization to the untreated CHO-S within each blot, BiP/GRP78 showed the clearest producer-associated response. Its abundance increased 1.7 ± 0.2-fold in CHO-S/HB8 and 2.3 ± 0.7-fold in 4BGD/DUL, compared with 1.2 ± 0.2-fold in CHO-S and 0.83 ± 0.05-fold in 4BGD. In contrast, the phospho-eIF2α response was modest and variable and did not show a clear producer-specific pattern.

CHOP/DDIT3 showed the strongest DTT response, with a several-fold increase in all four cell lines. The magnitude varied substantially between blots, mostly due to very low signal in some uninduced samples. Neither cleaved ATF6 (sATF6) nor calnexin showed a clear DTT-dependent change in abundance, and their levels were generally similar between treated samples across the four cell lines. Considerable variability in target-protein and GAPDH signals was observed among the three biological replicates. Therefore, Western blot quantification after normalization to GAPDH and to the untreated CHO-S sample was interpreted descriptively, and no inferential statistical comparisons were performed. Together, these data indicate that DTT-induced UPR outputs differed among the four cell lines, with the most consistent producer-associated effect being BiP induction in HB8 and DUL, whereas phospho-eIF2α, sATF6 and calnexin responses were more variable. 

### 2.3. Directional Enrichment Separates Induced Transport and Redox-Adaptation Programs from Repressed Stress-Signaling and Cell-State Networks

**Figure 4:**
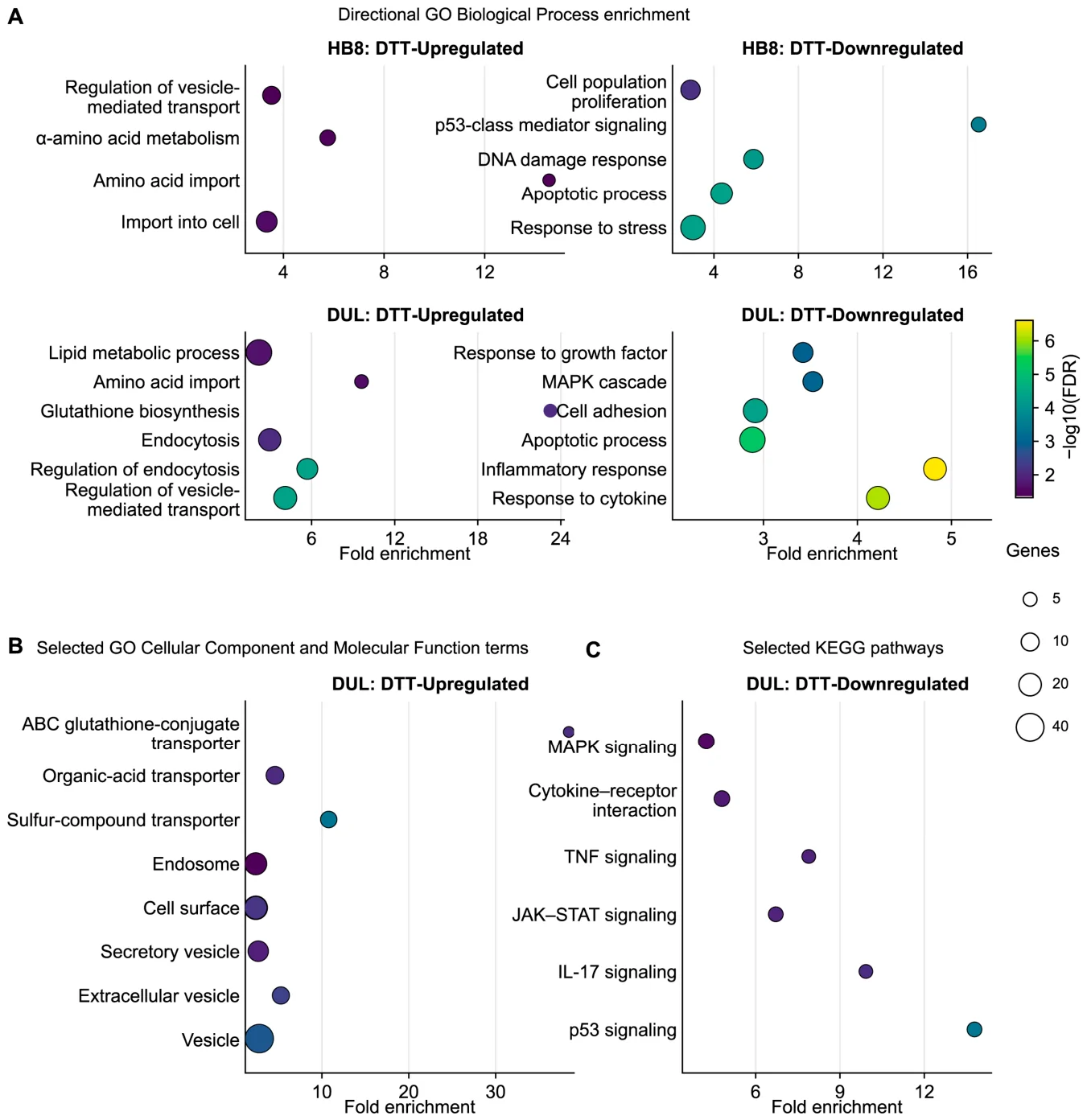
Directional enrichment analysis separates induced adaptive programs from repressed signaling and cell-state networks after DTT treatment. (**A**) Directional GO biological process enrichment analysis of genes significantly upregulated or downregulated after DTT treatment in HB8 and DUL producers. (**B**) Selected GO cellular component and molecular function terms enriched among DUL-upregulated genes. (**C**) Selected KEGG pathways enriched among DUL-downregulated genes. The x-axis shows fold enrichment, dot size the contributing gene count and dot color indicates statistical significance as −log10(FDR). Enrichment analyses were performed separately for upregulated and downregulated gene sets defined at adjusted *p*-value < 0.05 and |log2FC| > 1. Complete directional GO and KEGG enrichment results for the HB8 and DUL DTT responses are provided in Supplementary Dataset S4. Upregulated and downregulated refer to the DTT versus control comparison within each cell line. The differential-expression results shown in this figure and Figure 5 and Figure 6 were obtained from the HB8#3-excluded sensitivity dataset (2 × 3).

Detailed functional enrichment was performed using the HB8#3-excluded sensitivity dataset. These results were interpreted as exploratory pathway-level characterization, whereas the principal conclusion regarding the broader DUL response was based on its consistency with the complete three-replicate analysis. The same sensitivity dataset was used for analyses described below. Mixed functional enrichment analysis of the complete DTT-responsive gene sets initially indicated broad stress- and cell-state-associated remodeling, particularly in DUL. In HB8, the combined DE gene set was enriched for terms related to response to stimulus, stress, apoptotic signaling and cell death, together with selected extracellular and secretory-vesicle compartments. In DUL, the mixed analysis additionally identified cytokine- and inflammatory-response terms, adhesion and motility, vesicle-mediated transport and extracellular, cell-surface and vesicular compartments (Supplementary Dataset S3). However, because these analyses combined upregulated and downregulated genes, they did not distinguish transcriptionally induced programs from functional categories represented predominantly by repressed genes. We therefore analyzed the upregulated and downregulated fractions separately (Figure 4; Supplementary Dataset S4).

In HB8, the 276 genes upregulated after DTT treatment showed a relatively compact enrichment pattern (Figure 4A). Significant GO biological processes included import into the cell, amino-acid import across the plasma membrane, alpha-amino-acid metabolism and regulation of vesicle-mediated transport. Consistent with this pattern, the induced gene set contained *Slc7a11*, *Slc1a4* and *Gpt2*, together with ISR-associated markers such as *Trib3* and *Chac1* and iron-homeostasis-related genes including *Slc40a1* and *Fth1*. At the individual-gene level, this pattern is consistent with coordinated adjustment of amino-acid transport, glutathione-associated redox balance, iron handling and stress-associated metabolism. Thus, the induced HB8 response combined a transcriptional ISR-associated signature identified at the individual-gene level with coordinated changes in amino-acid transport, metabolism and selected vesicle-associated processes. In contrast, the 128 downregulated genes were strongly enriched for response to stress, apoptotic process, cell death, DNA-damage response, p53-associated signal transduction and regulation of signaling, with additional enrichment of cell-population proliferation and growth-factor-binding terms. This downregulated set included *Btg2*, *Bin3*, *Inka2*, *Sesn2*, *Ddit4*, *Errfi1*, *Mdm2* and *Plk2*. Several of these genes participate in immediate-early signaling, growth-factor-response feedback, cell-cycle control and stress-regulatory circuits, indicating that the repressed fraction includes regulatory nodes rather than a single coherent pro- or anti-apoptotic pathway. Therefore, the stress-, apoptosis- and cell-death-associated terms observed in the mixed HB8 analysis largely reflected repression of genes embedded in these regulatory networks rather than uniform transcriptional activation of an apoptotic program.

The directional separation was even more informative in DUL. Among the 743 DTT-upregulated genes, the most prominent enriched biological processes were regulation of vesicle-mediated transport, regulation of endocytosis, endocytosis, glutathione biosynthesis, amino-acid import and lipid metabolism (Figure 4A). Cellular-component enrichment further highlighted vesicles, extracellular vesicles, secretory vesicles, the cell surface and endosomes, whereas molecular-function analysis identified sulfur-compound, organic-acid and glutathione-conjugate transporter activities (Figure 4B). These results were consistent with induction of genes such as *Slc7a11*, *Slc1a4*, *Slc40a1*, *Fth1*, *Cat*, *G6pd*, *Trib3*, *Ddit3* and *Chac1*. The presence of *Cat* and *G6pd* additionally suggests engagement of antioxidant defense and NADPH-generating metabolism, complementing the glutathione- and transporter-associated enrichment detected at the pathway level. Thus, the induced DUL program combines ISR-associated transcriptional outputs with redox-related transport, antioxidant metabolic adaptation and extensive vesicle/endocytic remodeling.

By contrast, the 276 genes downregulated by DTT in DUL were enriched for a broad set of signaling and cell-state-associated processes (Figure 4A). Prominent GO terms included response to cytokine, inflammatory response, apoptotic process, cell death, cell adhesion, MAPK cascade and response to growth factor. The repressed gene set included *Btg2*, *Bin3*, *Errfi1*, *Inka2*, *Il33*, *Sesn2*, *Dusp6*, *Egr2* and *Ltbp1*. These genes include regulators of immediate-early transcriptional responses, MAPK feedback, growth-factor signaling and extracellular-matrix- or cytokine-associated communication, providing a gene-level counterpart to the broad signaling and cell-state categories identified by enrichment analysis. Consistent with the GO results, KEGG analysis of the DUL-downregulated fraction identified p53, IL-17, JAK-STAT, TNF, cytokine-cytokine receptor interaction and MAPK signaling pathways (Figure 4C). Extracellular-region and extracellular-matrix compartments, secretory vesicles and cell-cell junctions were also enriched, together with molecular functions related to signaling-receptor binding, kinase regulation and receptor-ligand activity. Thus, many of the cytokine-, inflammatory-, cell-death- and signaling-associated categories detected in the mixed DUL analysis originated predominantly from the downregulated component of the response rather than from transcriptional induction of these programs.

Overall, directional enrichment resolved the apparently mixed stress, cell-death and cytokine signatures obtained from the combined DE gene lists. The induced fractions in both producers contained evidence of amino-acid transport and metabolic adaptation together with ISR-associated marker genes, whereas DUL additionally showed prominent glutathione-related transport and vesicle/endocytic remodeling. Conversely, broad stress-, apoptosis-, cytokine-, growth-factor- and signaling-associated categories were concentrated in the downregulated fractions, particularly in DUL. These results indicate that DTT elicits an asymmetric transcriptional response consisting of induction of adaptive transport/redox programs together with repression and remodeling of pre-existing signaling and cell-state networks, rather than uniform activation of broad apoptosis or cytokine pathways.

### 2.4. HB8 and DUL Differ Strongly at Baseline and Retain Distinct Transcriptional States After DTT Treatment

**Figure 5:**
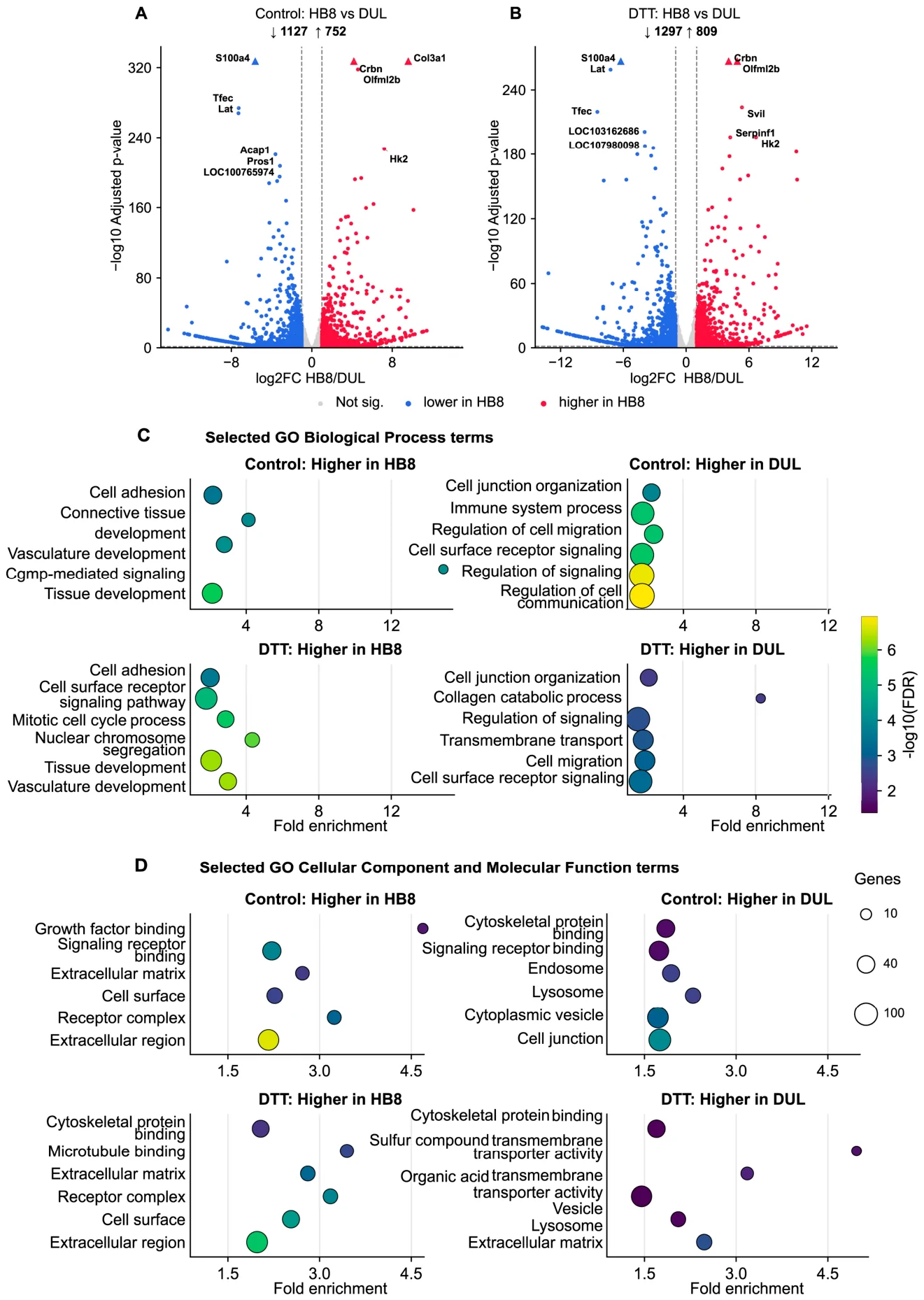
Interline transcriptional divergence and directional functional enrichment in HB8 and DUL under control and DTT-treated conditions. (**A**,**B**) Volcano plots comparing HB8 with DUL under control conditions and after DTT treatment, respectively. Positive log2FC values indicate higher expression in HB8 and negative values higher expression in DUL. (**C**) Selected gene ontology biological process terms significantly enriched among genes expressed at higher levels in HB8 or DUL under control and DTT-treated conditions. Directional gene sets were defined separately from the corresponding interline differential-expression comparisons, and representative non-redundant terms are shown. (D) Selected gene ontology cellular component and molecular function terms enriched among the same directional gene sets. For (**C**,**D**), the *x*-axis shows fold enrichment, bubble size indicates the number of genes associated with each term and color indicates −log10(FDR). Complete directional enrichment results underlying panels (**C**,**D**) are provided in Supplementary Dataset S5.

We next asked whether the broader DTT response observed in DUL occurred on a largely shared transcriptional background or was superimposed on pre-existing differences between the two producer lines. Direct comparison of untreated HB8 and DUL cells revealed extensive basal transcriptional divergence (Table 2, Figure 5A). In the complete three-replicate dataset, 1607 genes were differentially expressed between untreated HB8 and DUL cells. The HB8#3-excluded sensitivity analysis identified 1879 differentially expressed genes, including 752 genes expressed at higher levels in HB8 and 1127 genes expressed at higher levels in DUL. Thus, the two producers occupied markedly different transcriptional states before DTT exposure.

The interline divergence remained pronounced after DTT treatment (Figure 5B). In the complete three-replicate dataset, 1789 genes were differentially expressed between HB8 and DUL after DTT treatment. The corresponding HB8#3-excluded sensitivity analysis identified 2106 differentially expressed genes, including 809 genes expressed at higher levels in HB8 and 1297 genes expressed at higher levels in DUL. Of these interline differences, 1342 genes were significant in both untreated and DTT-treated comparisons, and all retained the same direction of change. These persistent genes accounted for approximately 71% of the baseline interline DE set and 64% of the DTT-state interline DE set. In addition, 537 genes were significant only under control conditions, whereas 764 genes were significant only after DTT treatment. Therefore, acute ER redox stress did not replace the pre-existing transcriptional identity of the two producers with a common stress state. Instead, a substantial line-specific component was preserved, while additional condition-dependent differences emerged after DTT exposure.

Directional functional enrichment further showed that the genes expressed at higher levels in HB8 and DUL represented distinct biological programs (Figure 5C,D; Supplementary Dataset S5). Under control conditions, HB8-high genes were enriched for biological processes related to tissue and connective-tissue development, vasculature-associated processes, cell adhesion and cGMP-mediated signaling, together with extracellular-region, extracellular-matrix, cell-surface and receptor-associated categories. Following DTT treatment, the HB8-high gene set additionally showed prominent enrichment of chromosome-segregation and mitotic cell-cycle processes and of microtubule- and cytoskeleton-associated molecular functions. In contrast, DUL-high genes under control conditions were enriched for regulation of cell communication and signaling, cell-surface receptor signaling, cell migration, immune-system-related processes and cell-junction organization, with associated enrichment of cell-junction, vesicular, lysosomal, endosomal and cell-surface compartments. After DTT treatment, DUL-high genes retained signaling-, migration- and cell-junction-associated enrichment and additionally showed terms related to transmembrane transport, collagen catabolism and extracellular-matrix remodeling, together with vesicle/lysosome-associated compartments and organic-acid- and sulfur-compound-transporter activities.

Complementary KEGG analysis of the complete interline DE sets provided an additional view of these differences (Supplementary Dataset S3). *Cytoskeleton in muscle cells*, *ECM–receptor interactions* and the *PI3K–Akt signaling pathway* were significantly enriched in both untreated and DTT-treated interline comparisons. In contrast, *Efferocytosis*, *Lysosome*, the *TGF-beta signaling pathway* and *Focal adhesion* reached FDR ≤ 0.05 only under control conditions. Thus, some pathway-level differences were maintained across treatment states, whereas others were more strongly associated with the untreated interline divergence.

Selected baseline interline differences also pointed to distinct ER proteostasis and stress-associated configurations. Among genes meeting the study-wide differential-expression criteria, *Eif2ak3*, *Trib3* and *Sqstm1* were expressed at higher levels in DUL, whereas *Pdia4*, *Ero1b* and *Creld2* were higher in HB8 (Supplementary Dataset S2). These examples suggested that the persistent interline divergence extended to stress-response, ER-folding and proteostasis-associated genes; these modules were examined systematically in the subsequent targeted analysis.

Overall, these results show that HB8 and DUL differ not only in the number of DTT-responsive genes but also in their underlying transcriptional organization. A substantial proportion of interline differences persists during acute ER stress, while DTT adds further state-dependent divergence rather than erasing producer-specific transcriptional identities.

### 2.5. Curated Secretory Pathway Modules Reveal Reciprocal Organization of ER Folding, ERAD, Redox and Stress-Survival Programs in HB8 and DUL

**Figure 6:**
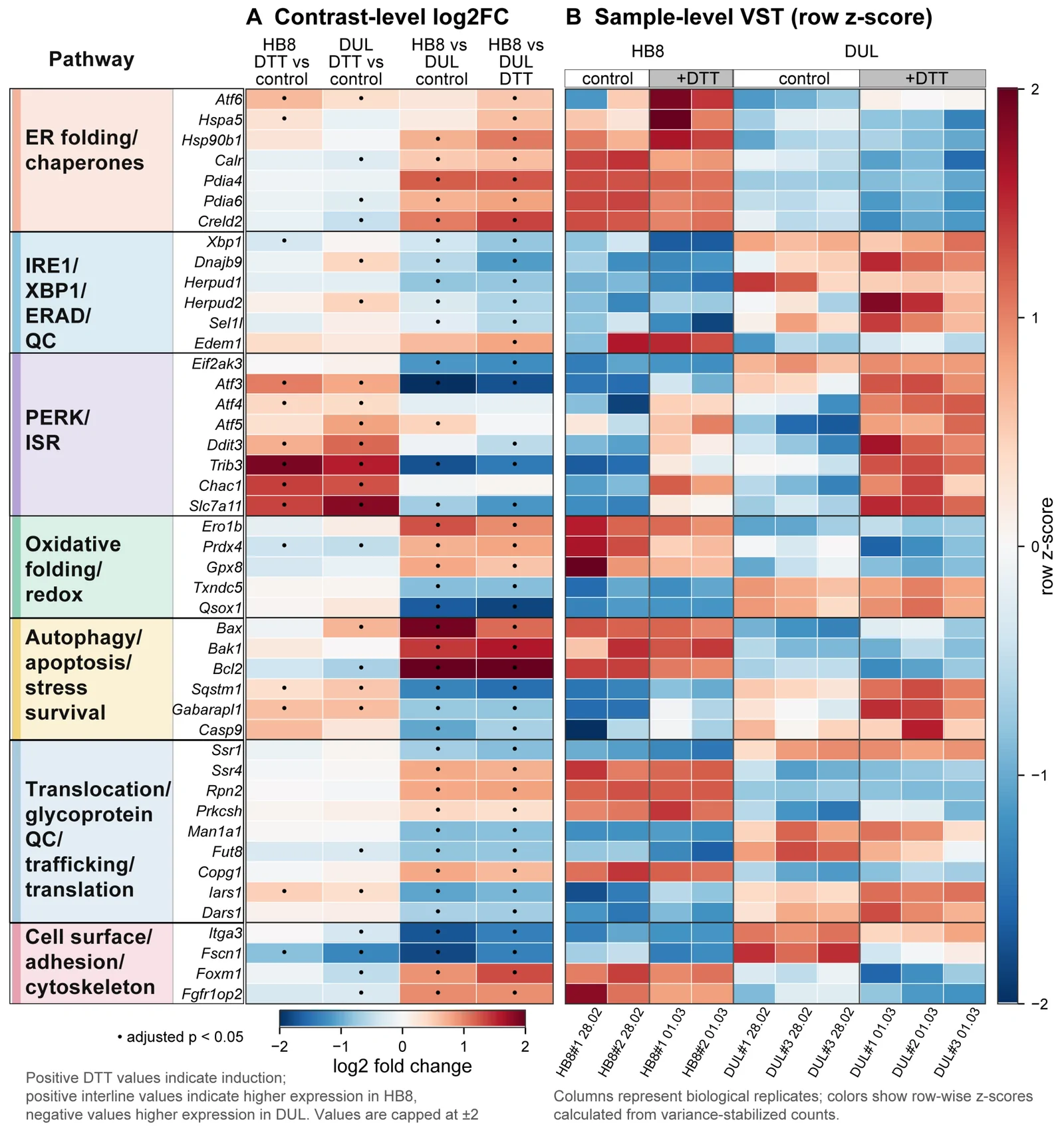
Curated ER proteostasis and secretory-pathway modules reveal reciprocal organization of folding, quality-control, redox and stress-survival programs in HB8 and DUL. (**A**) Contrast-level heatmap of selected genes from curated ER proteostasis, secretory-pathway and stress-survival modules. Columns show log2 fold changes for HB8 DTT versus HB8 control, DUL DTT versus DUL control, HB8 versus DUL under control conditions and HB8 versus DUL after DTT treatment. For DTT comparisons, positive values indicate induction after DTT treatment. For interline comparisons, positive values indicate higher expression in HB8 and negative values indicate higher expression in DUL. Dots indicate adjusted *p* < 0.05. For visualization, log2FC and row z-score values were capped at ±2. (**B**) Sample-level VST heatmap for the same selected genes. Values are row-wise z-scores calculated from variance-stabilized counts for individual biological replicates.

To complement broad GO and KEGG enrichment analyses, we performed a targeted inspection of predefined gene modules representing major components of ER proteostasis and the secretory pathway (Supplementary Table S1). This approach was intended to capture coordinated expression patterns that may be difficult to resolve by conventional enrichment analysis, particularly when individual genes show moderate but consistent differences. The curated set comprised 187 unique detected genes represented by 194 module assignments across 11 functional categories. These categories covered the major UPR branches, ER folding and redox functions, translocation, glycoprotein quality control, Golgi processing and vesicle trafficking. They also included translation and tRNA charging, autophagy, apoptosis, stress survival, cell surface adhesion and cytoskeletal remodeling. These modules were visualized both as a contrast-level log2FC map and as a sample-level VST (variance-stabilizing transformation) heatmap to distinguish consistent interline patterns from changes driven by individual comparisons (Figure 6A,B). Full-resolution versions of the heatmaps are provided in Supplementary Figure S1.

A prominent interline difference involved classical ER folding and oxidative-folding components. HB8 showed persistently higher expression of multiple chaperone- and folding-associated genes under both untreated and DTT-treated conditions, including *Pdia4*, *Creld2*, *Hsp90b1*, *Pdia6* and *Calr*. A similar HB8-high pattern was observed for several oxidative-folding and ER-redox genes, including *Ero1b*, *Prdx4* and *Gpx8*. In contrast, *Txndc5* and *Qsox1* were consistently higher in DUL. The VST heatmap confirmed that these differences were reproducible across individual samples rather than being driven by a single replicate. Thus, the redox/folding module was not shifted uniformly between the two producers but showed reciprocal organization, with HB8 retaining higher expression of several canonical ER folding and disulfide-forming factors and DUL expressing a distinct subset of thiol/redox-associated genes. Acute DTT treatment did not produce a coordinated transcriptional expansion of the entire chaperone or oxidative-folding machinery in either line. Notably, *Hspa5/BiP* increased only weakly at the transcript level in HB8 and was not transcriptionally induced in DUL, despite the protein-level BiP increase detected in both producer lines (Figure 3D,E).

The stress-signaling and ER quality-control modules showed a different organization. Several ISR-associated genes were expressed at higher levels in DUL both before and after DTT treatment, including *Eif2ak3*, *Atf3* and *Trib3*, with *Slc7a11* shifting further toward DUL after treatment. At the same time, the IRE1/XBP1/ERAD panel showed selective rather than uniform interline redistribution. *Xbp1*, *Dnajb9*, *Herpud1*, *Herpud2* and *Sel1l* were higher in DUL, whereas *Edem1* was higher in HB8. These patterns were also visible at the sample level in the VST heatmap. Thus, the curated data support a DUL-high subset of IRE1/XBP1-associated quality-control genes but do not indicate global elevation of the complete ERAD machinery. The DTT-induced *Xbp1* splicing described above (Figure 3B,C) further emphasizes that activation of the IRE1/XBP1 branch cannot be inferred from total *Xbp1* transcript abundance alone.

The autophagy/apoptosis/stress-survival module retained a strong signature of the engineered 4BGD background. *Bax* and *Bak1* transcripts were substantially lower in DUL than in HB8 both before and after DTT treatment, whereas *Sqstm1*, *Gabarapl1* and *Casp9* were higher in DUL. The annotated endogenous *Bcl2* transcript was, in contrast, higher in HB8. Because RNA-seq reads assigned to the endogenous hamster *Bcl2* locus do not provide a transgene-specific measurement, this observation should not be interpreted as evidence for absence or low expression of the introduced Bcl-2 cassette in 4BGD-derived cells. In our previous studies, it was shown that both Bcl-2 and Beclin-1 were downregulated following the *Bak1/Bax* double knockout. Their overexpression in the 4BGD cell line by stably integrated plasmids with EEF1A1 promoters restores both Bcl-2 and Beclin-1 levels according to the Western blot data. Overall, this module is consistent with persistent remodeling of apoptosis- and autophagy-associated gene expression in the DUL background rather than uniform upregulation of a generic stress-survival program.

Core translocation and early secretory-pathway genes were comparatively stable after acute DTT treatment. Most SRP, SEC61, signal-peptidase and COPI/COPII components showed modest within-line changes, although persistent interline differences were evident for selected genes. For example, *Ssr1* was higher in DUL, whereas *Ssr4* was higher in HB8. ER glycoprotein-quality-control genes likewise showed limited coordinated acute remodeling, but several stable line-specific differences were present, including higher *Rpn2* and *Prkcsh* expression in HB8 and higher *Man1a1* and *Fut8* expression in DUL. The VST data supported these as moderate but reproducible interline differences. In contrast, several low-abundance glycosylation-related genes with large apparent log2FC values, including *Mgat3* and *Galnt18*, were close to the lower expression range in the VST data and were therefore not used as primary markers in the main analysis. The translation/tRNA-charging panel showed a similarly modest acute DTT response, although several ribosomal protein genes were relatively higher in HB8, whereas selected aminoacyl-tRNA synthetases, including *Iars1* and *Dars1*, were higher in DUL. These patterns indicate that early secretory entry, glycoprotein processing, trafficking and translation are organized differently between the two producers but do not undergo uniform transcriptional activation after DTT exposure.

Finally, the curated cell-surface/adhesion/cytoskeletal module recapitulated persistent producer-specific differences at the level of individual genes. *Itga3* and *Fscn1* were higher in DUL under both untreated and DTT-treated conditions, whereas *Foxm1* and *Fgfr1op2* were higher in HB8. These reciprocal patterns were maintained across treatment states and provide a gene-level counterpart to the broader cell-surface, adhesion, vesicle-associated and cytoskeletal differences identified by interline enrichment analysis (Figure 5C,D).

Together, the curated module analysis indicates that HB8 and DUL differ in the internal organization of ER proteostasis and secretion-associated networks rather than along a single axis of stronger versus weaker UPR activation. HB8 showed persistently higher expression of multiple classical ER folding and oxidative-folding factors, whereas DUL was characterized by higher expression of selected ISR-associated, IRE1/XBP1-related quality-control, autophagy/stress-survival and cell-surface remodeling genes. Acute DTT exposure was superimposed on this pre-existing architecture: both producers activated stress-response programs, but neither showed uniform transcriptional expansion of the complete chaperone, ERAD, translocation or trafficking machinery. Thus, the broader DUL response is best interpreted as remodeling of a distinct pre-existing proteostasis and stress-survival state rather than as a uniformly stronger canonical UPR.

### 2.6. Transgene-Associated RNA Abundance Shows No Detectable Decrease During DTT-Induced ER Stress

**Figure 7:**
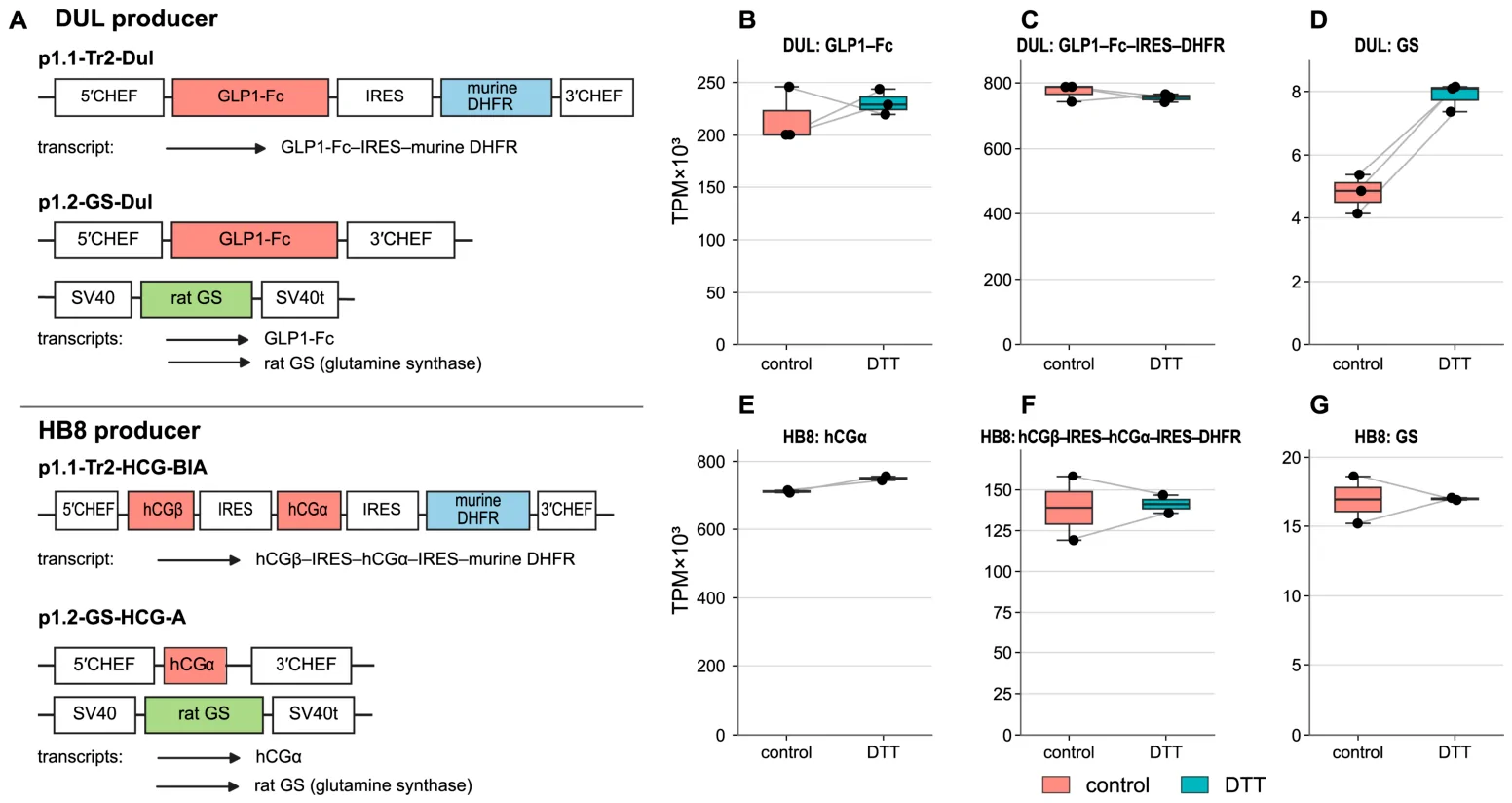
Transgene-associated transcript abundance during DTT-induced ER stress. (**A**) Schematic representation of the integrated heterologous expression cassettes analyzed in the two producer lines. The DUL producer contains the p1.1-Tr2-Dul construct encoding the polycistronic GLP1-Fc-IRES-murine DHFR transcript and the p1.2-GS-Dul construct containing separate 5′CHEF-GLP1-Fc-3′CHEF and SV40-rat GS-SV40t expression cassettes. The HB8 producer contains the p1.1-Tr2-HCG-BIA construct encoding the polycistronic hCGβ-IRES-hCGα-IRES-murine DHFR transcript and the p1.2-GS-HCG-A construct containing separate 5′CHEF-hCGα-3′CHEF and SV40-rat GS-SV40t expression cassettes. (**B**–**G**) Transgene-associated transcript abundance estimated by independent RNA-seq read mapping to an intron-free transgene-only reference. TPM values are shown for DUL GLP1-Fc (**B**), DUL GLP1-Fc-IRES-murine DHFR (**C**), DUL rat GS (**D**), HB8 hCGα (**E**), HB8 hCGβ-IRES-hCGα-IRES-murine DHFR (**F**) and HB8 rat GS (**G**). Each point represents one RNA-seq sample; grey lines connect matched control and DTT-treated samples from the same biological replicate. Boxes summarize the corresponding control and DTT groups. Y-axes start at zero to show transcript abundance relative to the full plotted range. Because transgene abundance was estimated by separate mapping to an artificial transgene-only reference, TPM values represent reference-specific transcript abundance and should not be interpreted as the fraction of total cellular mRNA.

To assess whether acute DTT treatment was associated with reduced steady-state RNA abundance from the integrated recombinant expression cassettes, we performed a separate transgene-directed RNA-seq analysis using an artificial reference composed of intron-free transgene sequences. All analyzed heterologous transcripts remained detectable after DTT treatment, and no significant treatment-associated difference in log2TPM (transcripts per million) was observed for any tested transgene target (Figure 7). This included the DUL GLP1-Fc transcript (*p* = 1.00), the GLP1-Fc–IRES–murine *Dhfr* transcript (*p* = 0.421), the *Gs* cassette (*p* = 0.421), the HB8 *hCGα* transcript (*p* = 0.548), and the polycistronic hCGβ–IRES–hCGα–IRES–murine *Dhfr* transcript (*p* = 0.690; Wilcoxon tests). Given the small number of biological replicates and the reference-specific nature of this analysis, these results were interpreted descriptively. Nevertheless, these results provide no evidence of a broad decrease in steady-state transgene-associated RNA abundance during DTT exposure. They do not establish whether recombinant-protein translation, folding, secretion, or specific productivity was maintained.

## 3. Discussion

Our data show that the CHO-S-derived HB8 producer and the CHO-4BGD-derived DUL producer occupy distinct pre-existing ER-proteostasis states and remodel them differently during DTT-induced reductive stress. HB8 showed higher expression of several classical ER folding and oxidative-folding factors, including Hsp90b1, Calr, Pdia3, Pdia4 and Ero1b. In contrast, DUL showed higher expression of selected ISR-associated, IRE1/XBP1-related quality-control and autophagy/stress-survival genes, including Eif2ak3, Trib3, Xbp1, Herpud1, Dnajb9 and Sqstm1. DUL also showed broader differences in vesicle- and cell-surface-associated programs.

Thus, the two producers do not differ along a simple axis of higher or lower ER capacity. Instead, they emphasize different components of proteostasis and stress adaptation. This supports the broader view that recombinant protein production is shaped by multiple secretory and stress-response bottlenecks rather than by transgene abundance alone [1,2]. However, HB8 and DUL also differ in host background, recombinant product, transgene architecture and clone-selection history. These patterns should therefore be interpreted as producer-specific associations rather than as effects caused solely by apoptosis resistance or the 4BGD genotype.

This producer-specific organization is consistent with the study by Malm et al., who identified host-dependent secretory-pathway differences and showed that co-expression of *Atf4*, *Srp9*, *Jun*, *Pdia3* or *Hspa8* could improve secretion of selected difficult-to-express proteins in CHO cells [2]. Several related genes were also informative in our dataset: *Atf4* was induced by DTT in both producers, whereas *Pdia3*, *Pdia4*, *Hspa8*, *Dnajc3* and *Ero1b* induction levels were higher in HB8. Together, these observations reinforce the idea that the limiting secretory module depends on the pre-existing state of the producer rather than on a universal high-secretion program.

Comparisons with Bai et al. and Sebastião et al. further support a context-dependent view of CHO producer adaptation. Bai et al. found that transgene copy number and product mRNA abundance were major determinants of productivity, whereas conserved transcriptional signatures of high productivity across CHO pools and clones were limited [4]. Sebastião et al. likewise showed that CHO clones producing a trispecific antibody differed in ER-stress, protein-synthesis, endocytic and degradation-associated programs according to product-quality phenotype [3]. In our dataset, several translation/tRNA-charging and vesicle-sorting-associated genes, including *Dars1*, *Iars1*, *Scfd1* and *Snx27*, also showed line-dependent expression. Although our study was not designed as a high- versus low-productivity or product-mispairing comparison, higher *Foxm1* expression in HB8 and higher *Fscn1* and *Itga3* expression in DUL similarly point to persistent differences in proliferation, cytoskeletal organization, adhesion and cell-surface state. Together, these studies support a model in which CHO producer phenotypes emerge from distinct combinations of secretory load, proteostasis capacity, trafficking organization and broader cell-state adaptation.

The origin of DUL from the engineered CHO 4BGD host provides a biologically plausible context for this distinct state. CHO 4BGD was directly derived from CHO-S and contains homozygous loss-of-function disruptions of Bax and Bak1. During development of this background, the BAX/BAK1 double-knockout precursor showed reduced BCL-2 and Beclin-1 protein abundance. Expression cassettes for BCL2 and BECN1 were subsequently introduced and restored their protein levels approximately to those observed in parental CHO-S cells, rather than producing a markedly supraphysiological BCL-2/Beclin-1 state [17]. The 4BGD background can therefore be viewed primarily as a productive CHO-S-derived cellular state with disabled BAX/BAK1-dependent apoptosis and compensatory normalization of BCL-2 and Beclin-1 abundance. Consistent with this history, DUL showed markedly higher expression of selected ISR-, ER quality-control- and autophagy-associated genes; differences in *Ssr1*, *Txndc5* and *Map1lc3b* further extended this pattern to secretory-pathway, redox and autophagy-related functions. These observations support the hypothesis that the 4BGD-derived producer occupies a stress-remodeled state in which apoptosis, autophagy and ER quality control are differently balanced. It should be noted that both *Bak1* and *Bax* transcripts in CHO-4BGD and DUL cells encode disrupted proteins due to frameshift mutations and premature stop codons. Thus, the observed *Bak*1/*Bax* transcript levels in DUL cells are not expected to result in functional proteins and should be interpreted as reflecting functionally inactive loci rather than the presence of intact pro-apoptotic effectors. Nevertheless, variation in their transcript abundance may remain biologically informative, as it could reflect the activity of upstream signaling pathways and transcriptional regulators controlling the intrinsic apoptotic program.

An important feature of the present study is that UPR activation was distributed across distinct regulatory layers. Both producers showed ISR/ATF4/CHOP-associated transcriptional outputs despite little change in *Eif2ak3*/*PERK* transcript abundance. Direct analysis of Xbp1 processing revealed strong DTT-induced splicing even though total *Xbp1* RNA-seq counts changed only weakly or decreased, demonstrating that activation of the IRE1/XBP1 branch was not captured by gene-level transcript abundance alone. Similarly, *Hspa5/BiP* showed limited transcriptional induction, particularly in DUL, whereas quantification of three immunoblots demonstrated reproducible BiP accumulation in both producer lines, with only weak or absent induction in the corresponding parental cells. This transcript–protein discordance is mechanistically compatible with the established post-transcriptional regulation of BiP during ER stress. BiP expression can be regulated at the level of translation efficiency, allowing protein synthesis to change without a proportional alteration in steady-state Hspa5 mRNA abundance [24]. Moreover, although eIF2α phosphorylation attenuates global translation during the integrated stress response, BiP synthesis can be maintained through non-AUG-initiated upstream open reading frames in the HSPA5 5′-UTR and the alternative initiation factor eIF2A [25]. These mechanisms provide a plausible explanation for the pronounced BiP accumulation observed despite the limited Hspa5 transcript response. Differences between the temporal profiles of Hspa5 transcription and BiP protein accumulation may also contribute to the discordance at the 24 h endpoint. However, the present experiments did not directly measure Hspa5 translation efficiency, ribosome occupancy, nascent BiP synthesis, or BiP turnover and therefore do not establish the relative contribution of these mechanisms in HB8 and DUL cells.

CHOP protein also increased several-fold in all four lines, although the magnitude varied markedly between blots due to very low signals in untreated samples. In contrast, eIF2α phosphorylation was modest and variable and did not show a clear producer-specific pattern, while sATF6 and calnexin levels remained broadly similar between treated samples. Together, these observations show that steady-state RNA abundance alone can underestimate branch-specific UPR activation and emphasize the value of integrating transcriptomic, RNA-processing and protein-level readouts. This discordance between pathway activation and transcriptional induction of upstream regulators is not unique to our system; transcriptomic analysis of tunicamycin-stressed potato similarly found extensive downstream ER-stress remodeling without early differential expression of several canonical ER-stress regulatory factors [26].

The preservation of transgene-associated RNA should likewise be interpreted strictly at the transcript level. PERK-dependent eIF2α phosphorylation can attenuate global translation during ER stress [27], while DTT can independently impair the folding and secretion of disulfide-containing proteins [28]. Therefore, unchanged transgene transcript abundance does not demonstrate preservation of recombinant-protein synthesis, secretion, or productivity. These outputs were not measured in the present experiment.

HB8 and DUL are not isogenic, and differences related to the recombinant product, transgene integration and clonal history cannot be completely excluded. Nevertheless, both lines are stable producers of secreted glycoproteins expressed from closely related plasmid systems, with comparable construct dimensions and transgene expression levels. Both products require ER translocation, disulfide-bond formation, glycosylation, folding and secretion, and their glycosylation burdens are of a comparable order. In contrast, the parental backgrounds differ in defined stress-response determinants, most notably the inactivation of BAX and BAK1 in CHO-4BGD. We therefore interpret the broader DUL response as being associated with its distinct host and producer background, in which the apoptosis-resistant genotype is a biologically relevant component, while recognizing that its individual causal contribution cannot be quantified without an isogenic comparison.

Directional enrichment clarified the biological meaning of the broader DUL response to DTT exposure. In both producers, induced genes were associated with amino-acid transport, metabolic adaptation and ISR markers, whereas DUL additionally activated glutathione-, antioxidant-, redox- and vesicle-associated programs. The concurrent induction of ISR markers and *Chac1* is consistent with coupling between the ATF4/ATF3-associated stress response and glutathione turnover, as CHAC1 is a stress-inducible glutathione-degrading enzyme transcriptionally regulated by ATF4 and ATF3 [29]. Similar links between sulfur-amino-acid availability, redox homeostasis, ER stress and culture performance have been reported in CHO fed-batch systems [11,12]. By contrast, cytokine-, inflammatory-, cell-death-, MAPK- and growth-factor-related terms were concentrated mainly among downregulated genes, particularly in DUL. Thus, these categories do not indicate broad activation of inflammatory or apoptotic programs. Rather, DTT elicited an asymmetric response in which adaptive transport and redox functions were induced while pre-existing signaling and cell-state networks were repressed or reorganized. The larger DUL response therefore reflects broader remodeling of an already distinct cellular state rather than uniformly stronger activation of the canonical UPR. 

The concurrent remodeling of amino-acid transport, redox metabolism and vesicle-associated processes in DUL may represent a functionally coupled adaptive program rather than three independent transcriptional outputs. Increased expression of amino-acid transporters, including Slc7a11 and Slc1a4, may support the supply and redistribution of substrates required for glutathione metabolism and stress-associated biosynthesis. Previous CHO studies have linked cysteine and cystine availability to glutathione balance, ER stress, cell viability and recombinant-protein production [11,12]. In parallel, induction of G6pd, Cat and Chac1 indicates reorganization of NADPH-dependent antioxidant capacity and glutathione turnover rather than simple enhancement of reductive buffering; CHAC1 itself is an ATF4/ATF3-regulated glutathione-degrading enzyme [29]. Vesicle-mediated transport and endocytic remodeling may complement these metabolic responses by adjusting membrane trafficking and protein-quality-control flux during impaired oxidative folding. Such coupling may be particularly relevant to the DUL clone examined here, with its apoptosis-resistant BAX/BAK1-deficient background representing one possible contributor. An extended stress-response phase could permit broader cellular remodeling before commitment to cell death. However, the present data demonstrate coordinated pathway engagement rather than direct biochemical synergy, and functional interactions among these modules remain to be tested experimentally.

DTT was used here as a defined perturbation of thiol-disulfide homeostasis to probe producer-specific vulnerability of redox-dependent ER folding, rather than as a direct mimic of fed-batch culture. Unlike tunicamycin and thapsigargin, which primarily disrupt N-linked glycosylation and ER Ca^2+^ homeostasis, respectively, DTT directly challenges oxidative folding and disulfide-bond formation, processes particularly relevant to recombinant glycoprotein production. The concentration of DTT and exposure time used in this study are comparable to conditions reported in previous studies and were not expected to cause significant cellular toxicity. In an independent follow-up experiment reproducing the same DTT concentration and exposure time, the calculated cytotoxicity index remained close to zero in both producer lines. Although these measurements were obtained from separately cultured samples rather than from the original RNA-seq cultures, they provide no evidence that the applied treatment regimen caused extensive cytolytic membrane damage.

Long-term fed-batch stress is more complex than acute DTT-induced stress and integrates nutrient limitation, mitochondrial dysfunction, glutathione imbalance and changing survival pressure. For example, Sinharoy et al. linked fed-batch cultivation of an industrial CHO producer to mitochondrial dysfunction, superoxide production, glutathione oxidation, ER-stress markers and product aggregation [10]. Additional CHO fed-batch studies have shown that manipulation of cysteine, cystine and tyrosine availability can alter glutathione/redox metabolism, ER-stress-associated pathways, viability and recombinant-protein productivity [11,12]. Thus, although acute DTT treatment differs fundamentally from prolonged production culture in both timescale and mechanism, it provides a controlled challenge for revealing producer-specific responses to impaired ER redox folding. Whether these signatures predict long-term fed-batch behavior requires validation using complementary stress models and direct sampling during fed-batch cultivation. However, transcriptional enrichment of glutathione metabolism and amino acid transport does not establish altered metabolite concentrations, pathway fluxes, or an improved intracellular redox state. Integrated transcriptomic and metabolomic analyses have demonstrated the value of directly relating cysteine availability and cellular stress responses to glutathione balance and productivity in CHO cells [11,30]. Future studies should therefore quantify intracellular amino acids, reduced and oxidized glutathione and related metabolites to determine whether the DUL-specific transcriptional response produces functional metabolic adaptation.

The present design did not include a recovery phase after DTT removal. Such a washout experiment would be informative regarding the reversibility of the DTT-induced state but would not directly reproduce prolonged fed-batch cultivation, in which the cellular environment remains dynamic and recombinant-protein production continues. Indeed, time-resolved analysis of an IgG1-producing CHO cell line demonstrated that UPR-associated transcriptional changes during fed-batch cultivation are dynamic and can be related directly to culture growth and production phenotypes [13]. The near-zero cytotoxicity index measured at the 24 h endpoint provides no evidence of extensive cytolytic membrane damage but does not establish long-term survival or preservation of productivity. Consequently, the broader transcriptional remodeling observed in DUL cannot be classified from the present data as either beneficial adaptation or dysregulated loss of homeostatic control. Distinguishing these alternatives will require time-resolved analysis under sustained stress and/or during fed-batch cultivation, including cell growth and viability, apoptosis, product titer, specific productivity and molecular markers of the UPR.

Taken together, our data support a model in which DTT acts on two pre-existing and transcriptionally distinct producer states rather than eliciting a uniform CHO-cell response (Table 3). We propose that the CHO-4BGD-derived DUL clone examined here occupies a distinct proteostasis state in which attenuation of the intrinsic apoptotic machinery may contribute to broader reorganization of ISR, ER quality-control, autophagy and stress-survival networks. Under acute reductive ER stress, this state does not produce uniformly stronger canonical UPR activation; instead, it is associated with extensive remodeling of redox metabolism, amino-acid transport, vesicle/endocytic pathways and broader cell-state programs. In contrast, the CHO-S-derived HB8 producer appears to rely more strongly on a pre-existing classical ER folding and oxidative-folding architecture and mounts a comparatively narrower transcriptional response. Thus, the major distinction between the two producers may lie not in the absolute strength of UPR activation, but in the organization of the proteostasis and stress-adaptation networks available before stress and recruited during the response.

This producer-state framework has practical implications for host-cell engineering. Previous studies have shown that manipulation of SRP-associated translocation components can improve secretion when early secretory entry is limiting, whereas XBP1s- and ERO1-related interventions can expand ER folding capacity or improve recovery from reductive stress in appropriate contexts [15,16]. The contrasting HB8 and DUL profiles suggest several candidate target classes for future testing. SRP/translocon components for early secretory entry; PDI-, ERO1-, TXNDC-, PRDX- and GPX-associated systems for redox-dependent folding. Additional candidates include XBP1- and ERAD-associated components for protein quality control and vesicle- or cytoskeleton-associated regulators of ER–Golgi and post-Golgi trafficking. Together, these results show that UPR activation in CHO producer cells is regulated at transcriptional, post-transcriptional and post-translational levels. BiP and CHOP accumulation, XBP1 splicing and eIF2α phosphorylation were not fully reflected by transcript abundance. Thus, transcriptomic analysis alone may underestimate UPR activation, supporting the use of integrated transcriptomic and protein-level approaches.

These transcriptomic signatures should be treated as hypotheses for targeted perturbation experiments rather than as evidence that individual genes are causal engineering targets. More broadly, our findings support a context-dependent engineering strategy in which the pre-existing proteostasis state of a producer is defined before selecting the secretory or stress-response module most likely to constrain performance in that background.

Several study limitations should be noted and taken into account for accurate data interpretation. The protein-level quantitative results should be interpreted cautiously because they were based on three biological replicates and normalization to GAPDH, whose stability under reductive stress cannot be assumed. In addition, because HB8 and DUL are non-isogenic producers, the individual contribution of the host background cannot be formally separated from product- and clone-associated effects. Nevertheless, both lines use closely related expression systems and produce secreted glycoproteins with broadly comparable biosynthetic demands; therefore, the distinct host background, including the BAX/BAK1-deficient 4BGD genotype, remains one biologically plausible contributor to the observed differences. The detailed HB8#3-excluded sensitivity analysis has a sample-size limitation because it includes only two matched HB8 biological pairs. Consequently, its dispersion estimates and exact threshold-based DEG counts should be interpreted cautiously. Nevertheless, the concordant PCA separation of both HB8#3 samples, the altered culture phenotype, their increased Cook’s distances and the alternative DUL#3 exclusion analysis provide independent support for examining the influence of this atypical pair in a separate sensitivity analysis.

A single atypical HB8 replicate pair is insufficient to demonstrate intrinsic transcriptomic instability. Because both untreated and DTT-treated HB8#3 samples were displaced, the observation also does not specifically indicate stress-induced instability.

Finally, no post-DTT recovery phase or time-resolved analysis under prolonged production conditions was included. Therefore, the present data do not establish the reversibility of the observed responses or their consequences for subsequent cell survival, growth and recombinant-protein productivity. Metabolite concentrations, metabolic fluxes and the intracellular GSH/GSSG balance were not measured.

## 4. Materials and Methods

### 4.1. Cell Lines

Four CHO cell lines were used in the DTT stress experiment: non-producing CHO-S cells (Thermo Fisher Scientific, Waltham, MA, USA), the previously developed apoptosis-resistant CHO 4BGD cells, the CHO 4BGD-derived dulaglutide analogue producer CHO 4BGD/Dul#73 (hereafter DUL) and the CHO-S-derived human chorionic gonadotropin producer CHO-S/HB8-Ags#18 (hereafter HB8).

CHO 4BGD, the parental host of DUL, contains homozygous disruptions of the pro-apoptotic genes *Bak1* and *Bax* and of the selection-marker genes *Dhfr* and *Glul*, together with genome-integrated expression cassettes with additional copies of *Bcl-2* and *Beclin-1* genes. Its generation, genomic characterization and phenotypic validation have been described previously [17].

DUL is a clonal producer of a dulaglutide analogue consisting of a GLP-1 peptide fused to the Fc fragment of human IgG4 and has been described previously [18].

HB8 is a clonal CHO-S-derived producer of human chorionic gonadotropin generated using Chinese hamster EF1α-based expression vectors, target-gene amplification, as described in [31], and subsequent cell cloning [32].

Both producer lines were selected for high productivity in clone-screening campaigns involving more than 200 candidate clones evaluated in static and fed-batch culture formats. ER-stress phenotypes were not used among the clone selection criteria.

### 4.2. Cell Cultivation and DTT Treatment

Cells were expanded for two passages in EmCD CHO 101 basal medium (Eminence, Suzhou, China) and used for experiments during logarithmic growth. For the parental non-producing CHO-S and CHO 4BGD cell lines, the medium was supplemented with L-glutamine (catalog no. Φ315; PanEco, Moscow, Russia) to a final concentration of 8 mM. No additional L-glutamine was added to the producer cell lines DUL and HB8, which carried integrated rat glutamine synthetase expression cassettes. Experimental cultures were established in 50 mL JET Biofil Bio-Reaction Tubes with vented caps (catalog no. BRT010050; JET Biofil/Guangzhou Jet Bio-Filtration Co., Ltd., Guangzhou, China) at an initial viable cell density of 1.0 × 10^6^ cells/mL in a working volume of 16 mL and maintained at 37 °C in a humidified 5% CO_2_ atmosphere with orbital shaking at 240 rpm on a 50 mm orbit. Viable cell density and viability were measured using a Countess II automated cell counter (Thermo Fisher Scientific, Waltham, MA, USA) and trypan blue exclusion.

For each cell line, three independent cultures were established as biological replicates. After 24 h of growth, viable cell density and viability were assessed, and untreated baseline samples were collected from each culture (T0). DTT treatment was initiated only in actively growing cultures with viability above 90%. A stock solution of 1 M DTT (dithiothreitol; molecular biology grade; catalog no. R0862; Fermentas/Thermo Scientific, Vilnius, Lithuania) was freshly prepared in water and added to a final concentration of 2 mM, and cells were incubated for a further 24 h under the same conditions. Post-treatment samples were collected from the same culture vessels at T24. 

Aliquots collected at T0 and T24 were used for RNA-based analyses and Western blotting. Cells were collected by centrifugation, washed once with PBS and divided into two aliquots of approximately 2 × 10^6^ cells each for RT-PCR assays: one aliquot of approximately 3 × 10^6^ cells for RNA-seq and one aliquot of approximately 5 × 10^6^ cells for Western blotting. After removal of residual PBS, all cell pellets were snap-frozen in liquid nitrogen and stored at −80 °C until processing.

### 4.3. LDH Release Assay

Plasma membrane integrity was assessed in an independent follow-up experiment using newly cultured HB8 and DUL cells. The original treatment conditions were reproduced, including exposure to 2 mM DTT for 24 h. The cultures used for the LDH assay were independent of those used for RNA-seq, Western blotting and the experiments presented in Figure 1. Extracellular LDH activity was measured in clarified culture supernatants using a kinetic assay at 340 nm according to the manufacturer’s instructions, LDH-2-OLVEX kinetic assay kit (Olvex Diagnostikum, St. Petersburg, Russia). Maximum LDH activity was determined after Triton X-100-mediated lysis of the corresponding cultures. LDH activities measured in Triton X-100-lysed samples were corrected for the 1.25-fold dilution introduced during lysis. Technical duplicate measurements were averaged before calculation. The cytotoxicity index was calculated separately for each biological replicate using the following equation:

Cytotoxicity index (%) = [(LDH_DTT − LDH_control)/(LDH_max,DTT − LDH_control)] × 100

where LDH_DTT is extracellular LDH activity in the DTT-treated culture, LDH_control is extracellular LDH activity in the corresponding untreated culture, and LDH_max,DTT is the dilution-corrected maximum LDH activity measured after Triton X-100 lysis of the DTT-treated culture. Three paired biological replicates were analyzed for each cell line. Negative calculated values were retained and indicate that extracellular LDH activity after DTT treatment did not exceed the spontaneous release measured in the corresponding untreated culture.

### 4.4. Western Blotting

Whole-cell lysates were prepared in modified RIPA buffer containing 50 mM Tris-HCl (pH 8.0), 150 mM NaCl, 1% Triton X-100, 0.1% sodium deoxycholate, 0.1% SDS, 1 mM EDTA and 0.01% NaN_3_. Immediately before use, the buffer was supplemented with 1 mM PMSF (Sigma-Aldrich, St. Louis, MO, USA), Antiase protease inhibitor cocktail and Antiphos AC/AL/S/T/Y phosphatase inhibitor cocktail (BioInnLabs, Rostov-on-Don, Russia) according to the manufacturer’s instructions. Cell pellets were lysed on ice for 30 min, and insoluble material was removed by centrifugation at 13,400 rpm for 10 min. Western blot experiments were performed using three independent biological replicates derived from separately cultured and DTT-treated cell samples. Each replicate was processed independently. 

Total protein concentrations were determined using the Micro BCA assay (Sigma), utilizing 2 mg/mL BSA solution (Sigma) as the protein concentration standard. Lysates were normalized by total protein concentration and mixed with SDS-PAGE loading buffer with 50 mM DTT. Unless otherwise stated, 5 µg of total protein was loaded per lane, using the 18-well comb. Proteins were resolved by 12% SDS-PAGE and transferred to nitrocellulose membranes (BioTrace™ NT, Pall Corporation, Port Washington, NY, USA) using a semi-dry system at 1 mA/cm^2^ for 2 h in Towbin transfer buffer containing 20% ethanol and no SDS.

Membranes were blocked for 1 h at room temperature in 5% non-fat dry milk in Tris-buffered saline containing 0.1% Tween-20 (TBST). After brief washing in TBST, membranes were sectioned according to molecular-weight range where required, and incubated overnight at 4 °C with primary antibodies diluted in 5% milk/TBST.

The following primary antibodies were used: anti-ATF6 (DF6009, Affinity Biosciences, Liyang, China; 1:1000), anti-phospho-eIF2α Ser51 (ARM2964, AssayVector, Katy, TX, USA; 1:1000), anti-eIF2α (E-AB-62069, Elabscience Biotechnology Inc., Wuhan, China; 1:1000), anti-DDIT3/CHOP (PAJ282Hu01, Cloud-Clone Corp., Wuhan, China; 1:1000), anti-HSPA5/BiP (MAC343Mu21, Cloud-Clone; 1:1000), anti-calnexin (MAA280Hu22, Cloud-Clone; 1:1000) and anti-GAPDH clone 6C5 (HyTest, Moscow, Russia; 1:10,000).

After washing with TBST (one 15 min wash followed by two 5 min washes), membranes were incubated for 1 h at room temperature with horseradish peroxidase-conjugated secondary antibodies: goat anti-rabbit IgG(H+L)-HRP (E-AB-1194, Elabscience, China; 1:2000) or anti-mouse IgG-HRP (ab6789, Abcam, Waltham, MA, USA; 1:2000), as appropriate. Signals were developed using Pierce ECL Western Blotting Substrate (Thermo Fisher Scientific, Waltham, MA, USA) and recorded with a Fusion FX imaging system (Vilber Lourmat, Collégien, France).

Densitometric analysis was performed using TotalLab software (version 2009; Nonlinear Dynamics Ltd., Newcastle upon Tyne, UK). Band intensities were normalized to GAPDH. Because GAPDH abundance may vary under cellular stress, the normalized Western blot results were interpreted descriptively. No consistent DTT-dependent change in GAPDH signal was observed across the four cell lines. Samples from biological repeat 1 were also loaded as 4 µg of total protein per lane, resolved by 12% SDS-PAGE, stained with colloidal Coomassie blue and scanned on a flatbed scanner in transparent mode for visual assessment of sample uniformity (Supplementary Dataset S1).

### 4.5. XBP1 Splicing Assay

Activation of the IRE1/XBP1 branch of the unfolded protein response was assessed by semi-quantitative RT-PCR analysis of Xbp1 mRNA splicing. Total RNA was isolated using the GeneJET RNA Purification Kit (Thermo Fisher Scientific, Waltham, MA, USA). RNA concentration was measured using a Qubit fluorometer with the RNA BR Assay Kit (Invitrogen, Carlsbad, CA, USA), and cDNA was synthesized using the Mint-2 cDNA synthesis kit (Evrogen, Moscow, Russia).

PCR amplification was performed using primers flanking the unconventional IRE1-dependent splice site in the Xbp1 transcript: Xbp1-F, 5′-CTCGCTTGGGAATGGATGTG-3′, and Xbp1-R, 5′-GGTAGACCTCTGGGAGTTC-3′. This primer pair amplifies both unspliced and spliced Xbp1 isoforms, yielding products of 305 bp for unspliced Xbp1 (Xbp1u) and 279 bp for spliced Xbp1 (Xbp1s).

PCR was performed using ScreenMix-HS PCR mix (Evrogen, Moscow, Russia) with an initial denaturation at 95 °C for 3 min, followed by 35 cycles of 95 °C for 10 s, 58 °C for 10 s and 72 °C for 15 s. Amplification products were separated by electrophoresis in 1.8% agarose gels prepared in TBE buffer and visualized by ethidium bromide staining.

Band intensities were quantified using TotalLab software (version 2009; Nonlinear Dynamics Ltd., Newcastle upon Tyne, UK). The relative XBP1 splicing level was calculated as described by Yoon et al. [23] as the proportion of the spliced product in the combined spliced and unspliced Xbp1 signal: XBP1 splicing (%) = Xbp1s/(Xbp1s + Xbp1u) × 100%.

### 4.6. RNA-Seq Sample Quality Control, Library Preparation and Sequencing

Total RNA from untreated and DTT-treated DUL and HB8 cultures was subjected to transcriptome sequencing. The RNA-seq experiment comprised three paired biological replicates per producer line, with control and DTT-treated samples obtained from the same replicate cultures as described above. RNA quality assessment, library preparation and sequencing were performed by BGI Hong Kong Tech Solution NGS Lab.

RNA quantity and integrity were assessed before library preparation using a Fragment Analyzer. All submitted samples met the sequencing provider’s quality criteria for library construction. Reported RNA integrity values ranged from 6.7 to 10.0, RNA concentrations from 312 to 1850 ng/µL and total RNA amounts from 9.36 to 55.5 µg.

Strand-specific mRNA libraries were prepared using the DNBSEQ Eukaryotic Strand-specific Transcriptome Resequencing workflow. Polyadenylated mRNA was enriched from total RNA using oligo(dT) beads and fragmented. First-strand cDNA was synthesized using random primers, followed by second-strand synthesis with dUTP in place of dTTP to preserve strand specificity. The resulting cDNA fragments underwent end repair, 3′ adenylation, adaptor ligation, PCR amplification and library quality control. Libraries were subsequently circularized and amplified to generate DNA nanoballs (DNBs) for sequencing on the DNBSEQ platform.

Sequencing was performed in paired-end 150-bp mode (PE150). Each sample yielded 10.80–10.85 Gb of clean sequence data. Across libraries, Q20 and Q30 base proportions ranged from 98.11% to 98.44% and from 93.36% to 94.45%, respectively, and GC content ranged from 46.85% to 48.68%. Clean FASTQ files were provided with Phred+33 quality encoding.

### 4.7. RNA-Seq Data Processing and Differential Expression Analysis

Raw sequencing data were initially filtered by BGI using SOAPnuke. Adapter-containing reads, contaminating reads, polyX-containing reads and low-quality reads were removed. The SOAPnuke filtering parameters were: ‘-n 0.01 -l 20 -q 0.4 --adaMR 0.25 --polyX 50 --minReadLen 150’. Reads were discarded if 25% or more of the read matched adapter sequence, allowing a maximum of two base mismatches; if the read length was less than 150 bp; if the N content exceeded 1% of the read; if a polyX stretch exceeded 50 bp; or if bases with quality score below 20 accounted for 40% or more of the read. Clean reads were provided in Phred+33 FASTQ format.

Clean sequencing data were additionally evaluated using FastQC version 0.12.1. Adapter sequences and low-quality nucleotides were removed using Trimmomatic version 0.40. Adapter trimming was performed with the ILLUMINACLIP option using parameters 2:30:10. The first 15 bp of each read were removed using HEADCROP:15. Low-quality bases at read ends were removed using LEADING:3 and TRAILING:3. A sliding window of 4 bp with an average quality threshold of 20 was applied, and reads shorter than 36 bp after trimming were excluded.

Processed reads were aligned to the Cricetulus griseus reference genome CriGri-PICRH-1.0, assembly GCF_003668045.3, using HISAT2 version 2.2.2 [33] with default parameters. The corresponding gene annotation file was used for downstream gene-level quantification. Alignment files were processed using SAMtools version 1.9 [34]. Read counts per gene were generated using featureCounts version 2.0.6 [35]. Genes with a total count of fewer than 10 reads across all libraries were excluded from further analysis. Library-specific sequencing-quality, trimming, alignment and gene-assignment statistics are provided in Supplementary Table S2.

Sample relationships were assessed by principal component analysis of variance-stabilized expression data together with the available culture-phenotype and library-quality information. Both samples of the HB8#3 matched pair showed concordant separation from the remaining HB8 samples along PC2 and originated from a culture with lower viable cell density and viability. 

Differential expression was first assessed using the complete dataset comprising three matched biological replicate pairs per producer. To evaluate the influence of the atypical HB8#3 culture, the complete workflow was repeated after exclusion of this matched pair. The complete dataset was used for the primary comparison of response magnitude, whereas the HB8#3-excluded dataset was treated as a secondary sensitivity analysis and used for detailed gene- and pathway-level exploration. The decision to examine the HB8#3-excluded dataset was made during initial unsupervised quality control, before inspection of DEG numbers or identities.

Differential expression analysis was performed using DESeq2 version 1.49.3 [36] in R. The main comparisons were as follows: HB8 DTT versus HB8 untreated control, DUL DTT versus DUL untreated control, HB8 untreated control versus DUL untreated control and HB8 DTT versus DUL DTT. For within-line DTT comparisons, biological replicate pairing was included in the design matrix where applicable. For between-line comparisons, samples were compared within the same treatment condition. Genes were considered differentially expressed if they had an adjusted *p*-value < 0.05 and an absolute log2 fold change > 1. Genes not passing these thresholds were retained for targeted inspection of predefined UPR, ER stress and secretory pathway markers.

To assess whether the change in differential-expression results following exclusion of HB8#3 could be explained solely by reducing the number of biological replicates from three to two, an additional sensitivity analysis was performed by alternatively excluding the DUL#3 matched pair. Unlike HB8#3, DUL#3 did not show anomalous separation in the PCA and was therefore used as a non-outlying control exclusion. The complete DESeq2 workflow, including normalization and dispersion estimation, was rerun for three datasets: all samples, the dataset excluding HB8#3 and the dataset alternatively excluding DUL#3. The same statistical thresholds were applied in all analyses: an adjusted *p*-value < 0.05 and an absolute log2 fold change > 1. This sensitivity analysis was performed to evaluate the effect of sample composition and was not used for further sample selection.

The sequencing reads generated in this study have been deposited in the NCBI Sequence Read Archive (SRA) under BioProject accession PRJNA1492564.

### 4.8. Functional Enrichment Analysis

Functional enrichment analysis was performed for differentially expressed gene sets using Gene Ontology Biological Process, Cellular Component and Molecular Function categories and KEGG pathway annotations. Enrichment analysis was carried out using ShinyGO 0.85.1 [37]. *p*-values were adjusted for multiple testing using the Benjamini–Hochberg method. Terms with FDR < 0.05 were considered significant. For exploratory KEGG analysis, terms with FDR < 0.1 were additionally inspected and reported as trends where indicated. Complete mixed GO and KEGG enrichment results are provided in Supplementary Dataset S3. Directional enrichment results for the within-line DTT responses and interline comparisons are provided in Supplementary Datasets S4 and S5, respectively. Functional enrichment analyses reported in Figure 4, Figure 5 and Figure 6 were performed using differential-expression results from the HB8#3-excluded sensitivity dataset (2 × 3).

To support interpretation of ER stress and secretory pathway responses, curated gene panels were assembled for the following functional modules: PERK/ISR, IRE1/XBP1/ERAD, ATF6/chaperones, oxidative folding/disulfide formation, ER translocation and early secretory pathway, Golgi glycosylation, vesicle trafficking and autophagy/apoptosis/stress survival (Supplementary Table S1). The initial curated panel comprised 193 unique genes represented by 200 module assignments; six genes were not represented in the final filtered RNA-seq matrix and were excluded from heatmap visualization but retained in the supplementary curated list. Heatmaps were generated from either normalized expression values or log2 fold changes, as indicated in figure legends.

### 4.9. Transgene Expression Analysis

Expression of integrated heterologous expression cassettes was evaluated separately from the host-transcriptome analysis. Both producer lines had been generated using pairs of expression plasmids based on Chinese hamster elongation factor 1 alpha regulatory elements and contained integrated cassettes encoding the corresponding genes of interest together with selectable markers. The analyzed transgene-derived transcripts included target-gene sequences linked through internal ribosome entry sites (IRESs) to murine *Dhfr*, target-gene transcripts expressed from separate EF1α-based cassettes and rat glutamine synthetase (*Gs*) selection cassettes expressed from an independent SV40 promoter.

To minimize ambiguous assignment of reads from sequences homologous to the hamster genome, transgenes were analyzed separately from the primary host-transcriptome reference. Candidate transgene regions were screened against the CriGri-PICRH-1.0 genome by BLAST+ v2.17.0, and Chinese hamster intronic sequences present in the original constructs were excluded. An artificial reference containing intron-free transgene sequences was then generated, and transcript abundance was quantified as transcripts per million (TPM) using Salmon v. 2.2.1 [38] in quasi-mapping mode. TPM values were log2-transformed where indicated.

This analysis was intended as a reference-specific, descriptive estimate of transgene-associated transcript abundance rather than as part of the host-gene differential-expression framework. Accordingly, transgene counts were not analyzed with DESeq2. Control and DTT-treated values were examined as matched biological-replicate pairs. Because of the small number of paired samples, the analysis was interpreted primarily descriptively.

## 5. Conclusions

HB8 and DUL occupy distinct ER-proteostasis states and mount quantitatively and functionally different responses to DTT-induced reductive stress. The CHO-S-derived HB8 producer shows a more folding- and redox-oriented basal profile and a narrower transcriptional response, whereas the CHO-4BGD-derived DUL producer displays broader transcriptional remodeling associated with redox regulation, amino acid transport, vesicular trafficking and cell-state programs. These differences may reflect the combined influence of the distinct host and producer backgrounds. The apoptosis-resistant BAX/BAK1-deficient 4BGD genotype represents one biologically plausible contributor, but its contribution cannot be separated from product-, transgene- and clone-associated effects in the present non-isogenic design. Integration of RNA-seq with XBP1-splicing and protein-level analyses further shows that UPR activation cannot be inferred from transcript abundance alone. No detectable decrease in transgene-associated RNA abundance was observed during DTT treatment, but recombinant-protein synthesis, secretion and productivity were not assessed. Together, these findings support a host- and producer-state model of acute ER-stress adaptation and identify candidate pathways for functional validation and future engineering of stress-resilient CHO cells.

## Figures and Tables

**Table 1:** Summary of differential expression analysis across DTT-response.

Group 1	Group 2	Significant DE Genes (3 × 3)	Significant DE Genes (2 × 3)	Up in Group 1 (2 × 3)	Up in Group 2 (2 × 3)	Observation
HB8_DTT	HB8_control	299	404	276	128	Moderate DTT-induced response in the CHO-S-derived producer
DUL_DTT	DUL_control	877	1019	743	276	Broader DTT-induced response in the 4BGD-derived producer

Note. Differential expression was assessed using DESeq2. Genes were considered significant at adjusted *p* value < 0.05 and absolute log2 fold change > 1; “up in group 1” corresponds to genes upregulated after DTT treatment.

**Table 2:** Summary of differential expression analysis across interline comparisons.

Group 1	Group 2	Significant DE Genes (3 × 3)	Significant DE Genes (2 × 3)	Up in Group 1 (2 × 3)	Up in Group 2 (2 × 3)	Observation
HB8_control	DUL_control	1607	1879	752	1127	Strong basal transcriptional divergence between producer backgrounds
HB8_DTT	DUL_DTT	1789	2106	809	1297	Interline differences persist and expand after DTT treatment

Note. Differential expression was assessed using DESeq2. Genes were considered significant at adjusted *p*-value < 0.05 and absolute log2 fold change > 1. “Up in group 1” corresponds to genes higher in HB8, whereas “up in group 2” corresponds to genes higher in DUL.

**Table 3:** Comparative summary of basal proteostasis organization and DTT-induced remodeling in HB8 and DUL producers.

Feature	HB8	DUL
**Basal state**	Higher classical ER folding and oxidative-folding factors	Higher expression of selected ISR-, ER quality-control- and stress-survival-associated genes disrupted *Bax* and *Bak1* loci
**DTT response breadth**	Narrower	Broader
**Major induced programs**	ISR-associated core; amino-acid and metabolic adaptation	ISR-associated response; glutathione/redox, amino-acid, vesicle and endocytic remodeling
**UPR activation**	*Xbp1* splicing, BiP protein response and eIF2α phosphorylation despite limited transcript-level changes	*Xbp1* splicing, BiP protein response and eIF2α phosphorylation despite weak *Hspa5* and total *Xbp1* transcript induction
**Overall interpretation**	Folding/redox-oriented proteostasis state	Stress-remodeled state with broader network reorganization

## Data Availability

The sequencing reads generated in this study are openly available in the NCBI Sequence Read Archive (SRA) under BioProject accession PRJNA1492564. Processed count matrices, differential-expression results and curated gene-module tables are provided in the Supplementary Materials.

## Supplementary Materials

The following supporting information can be downloaded at: https://www.mdpi.com/article/10.3390/ijms27167427/s1 [39,40,41,42,43,44,45,46,47,48,49].

## Abbreviations

The following abbreviations are used in this manuscript:

ATF4	Activating transcription factor 4
CHOP	C/EBP homologous protein
CHO	Chinese hamster ovary
DTT	Dithiothreitol
ER	Endoplasmic reticulum
ERAD	Endoplasmic-reticulum-associated degradation
FDR	False discovery rate
GO	Gene Ontology
IRE1	Inositol-requiring enzyme 1
ISR	Integrated stress response
KEGG	Kyoto Encyclopedia of Genes and Genomes
PCA	Principal component analysis
PERK	Protein kinase RNA-like endoplasmic reticulum kinase
RNA-seq	RNA sequencing
RT-PCR	Reverse-transcription polymerase chain reaction
TPM	Transcripts per million
UPR	Unfolded protein response
VST	Variance-stabilizing transformation
XBP1	X-box-binding protein 1
