# Supplementary material for: DTT-Induced Transcriptome Profiling Reveals Divergent ER Proteostasis and Stress-Adaptation Programs in CHO-S/HB8 and CHO-4BGD/DUL Producer Clones
Source: Int J Mol Sci. 2026 Aug 19;27(16):7427. doi: 10.3390/ijms27167427 (PMC13513848; doi:10.3390/ijms27167427)
Supplement: Supplementary file 1 [file ijms-27-07427-s001.zip › Blotting membranes description.pdf]

Supplementary File Sx

Source data and membrane description for Figure 3D and Figure 3E

Figure 3D contains densitometric quantification of the Western blot signals shown in Figure 3E. Figure 3E is a composite Western blot panel assembled from six original image files corresponding to independently processed membranes or membrane halves. Some membranes were cut into upper and lower halves and probed separately with different antibodies. BiP and GAPDH were probed simultaneously using two mouse primary antibodies. All membranes and membrane halves were prepared using the same lysate set and loaded in the same lane order.

Source images for the Figure 3E:

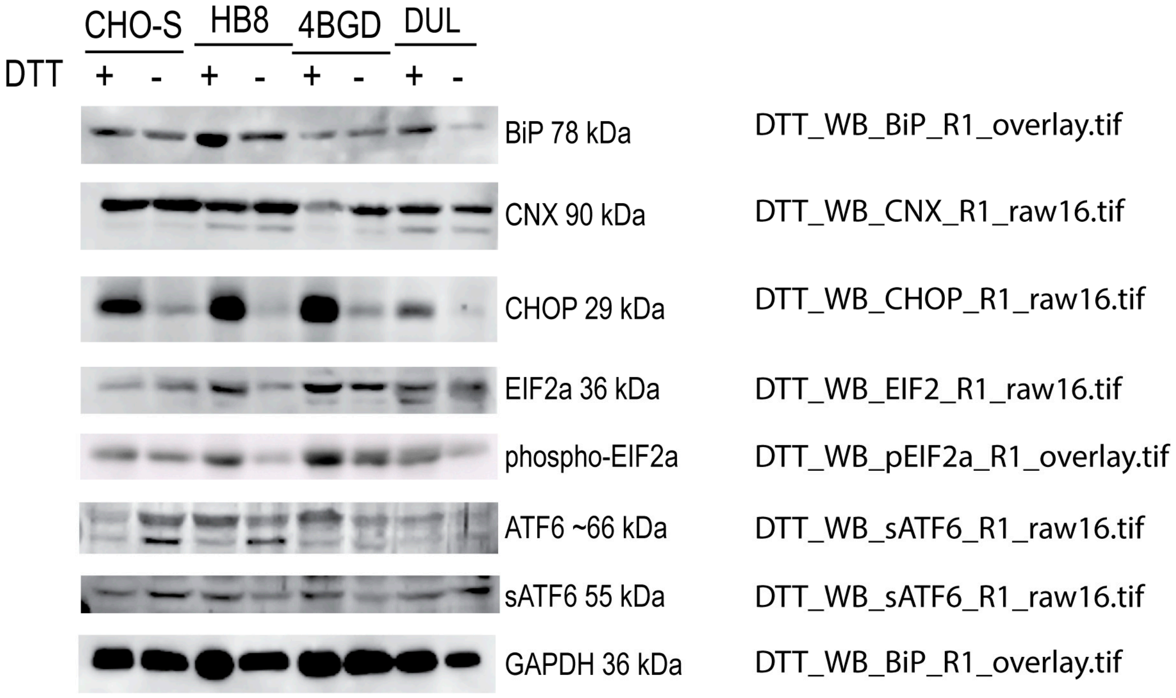

DTT_WB_BiP_R1_overlay.tif

DTT_WB_CNX_R1_raw16.tif

DTT_WB_CHOP_R1_raw16.tif

DTT_WB_EIF2a_R1_overlay.tif

DTT_WB_pEIF2a_R1_overlay.tif

DTT_WB_sATF6_R1_raw16.tif

DTT_WB_sATF6_R1_raw16.tif

DTT_WB_BiP_R1_overlay.tif

Additional loading/sample-quality control

A Coomassie-stained SDS-PAGE gel loaded with the same lysate preparations and the same lane order is provided as an additional sample loading and protein-profile control. This gel was not used for densitometric quantification in Figure 3D. The gel was loaded at 4 µg/lane.

Original file: Coomassie staining.tif
