# Supplementary material for: DTT-Induced Transcriptome Profiling Reveals Divergent ER Proteostasis and Stress-Adaptation Programs in CHO-S/HB8 and CHO-4BGD/DUL Producer Clones
Source: Int J Mol Sci. 2026 Aug 19;27(16):7427. doi: 10.3390/ijms27167427 (PMC13513848; doi:10.3390/ijms27167427)
Supplement: Supplementary file 1 [file ijms-27-07427-s001.zip › DTT-IJMS-Figure S1_v2.pdf]

Figure S1. Full heatmap of curated ER-proteostasis, secretory-pathway and stress-survival modules.

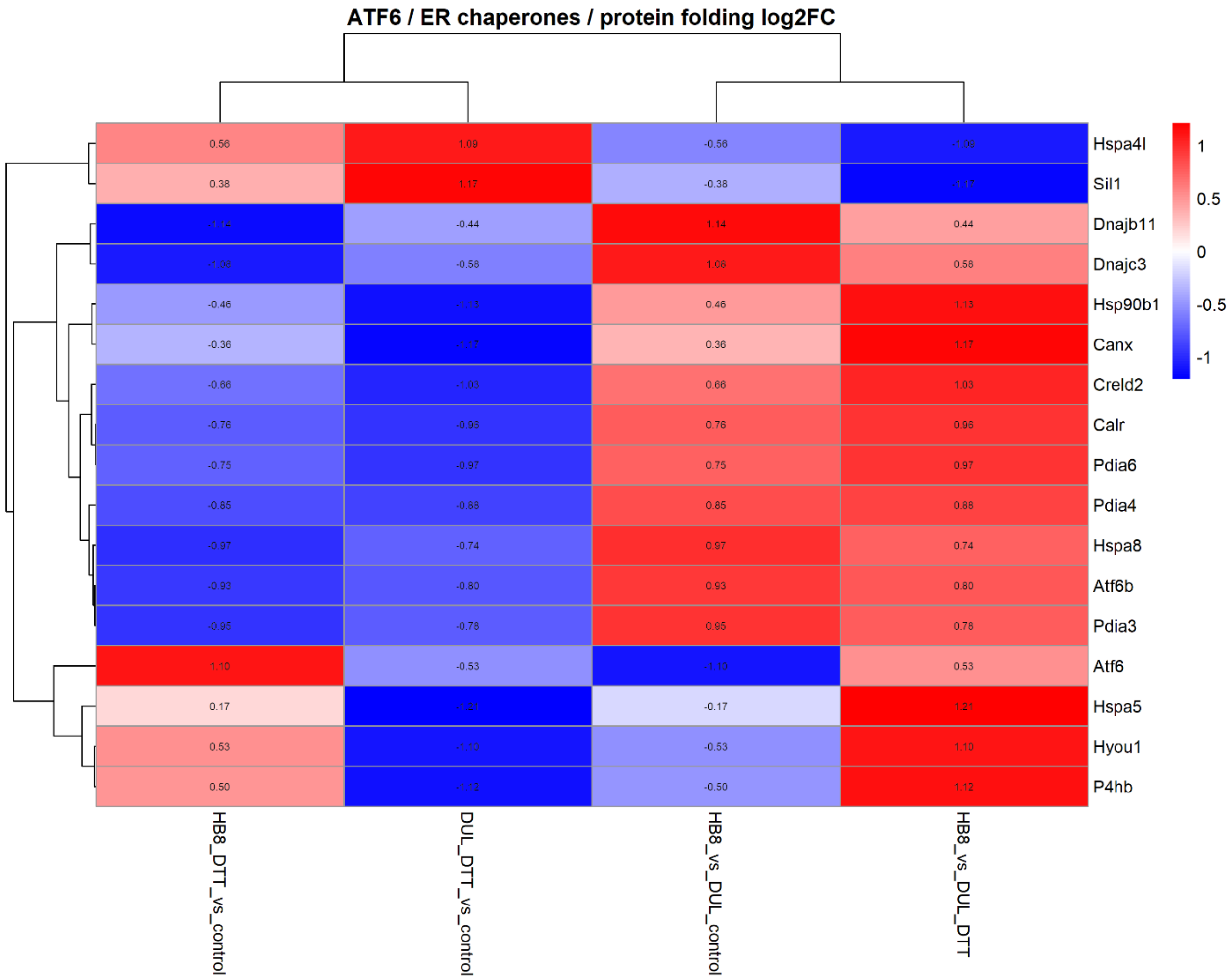
ATF6 / ER chaperones / protein folding log2FC
Hspa4l
Sil1
Dnajb11
Dnajc3
Hsp90b1
Canx
Creld2
Calr
Pdia6
Pdia4
Hspa8
Atf6b
Pdia3
Atf6
Hspa5
Hyou1
P4hb
HB8_DTT_vs_control
DUL_DTT_vs_control
HB8_vs_DUL_control
HB8_vs_DUL_DTT
1
0.5
0
-0.5
-1

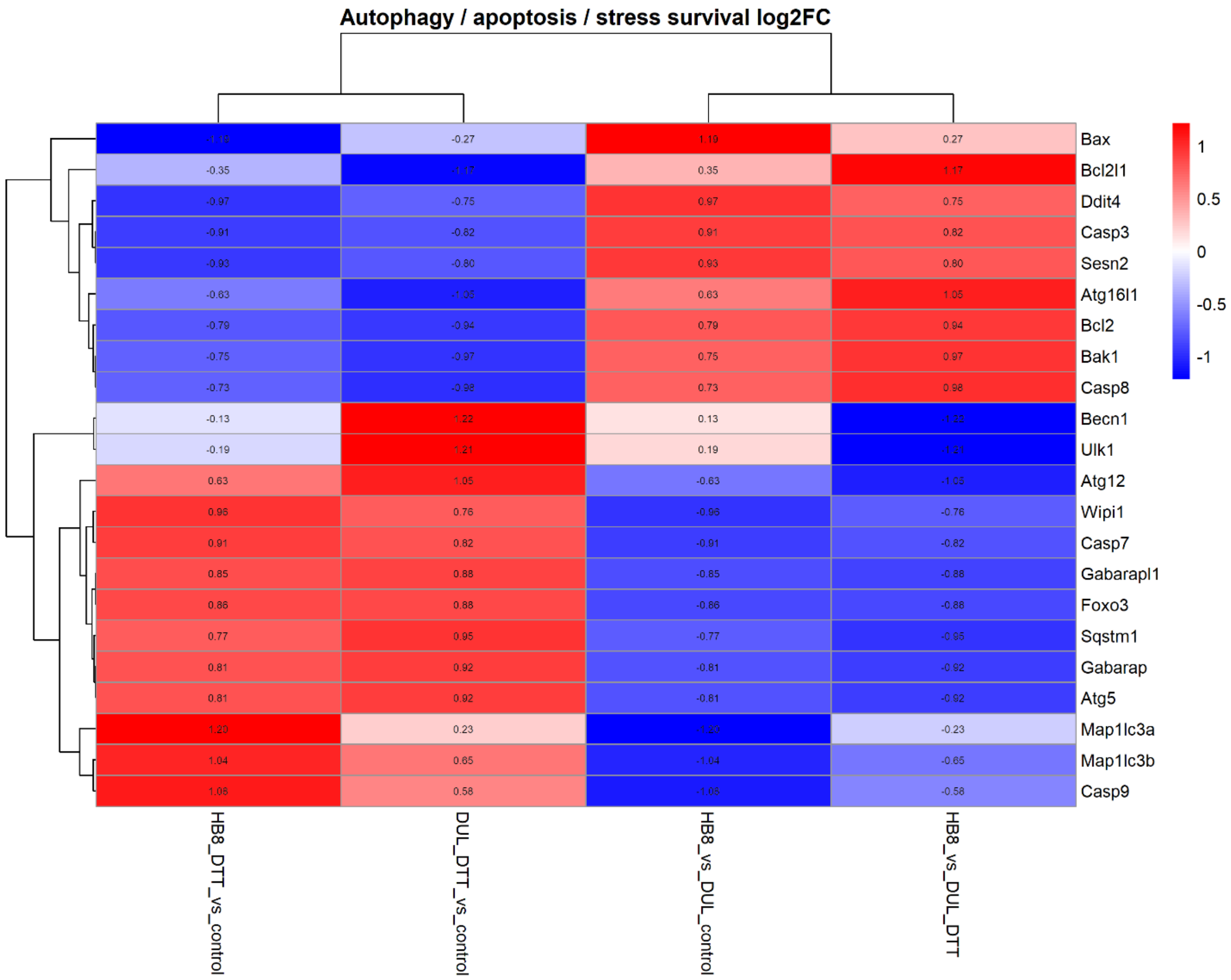
Autophagy / apoptosis / stress survival log2FC
Bax
Bcl2l1
Ddit4
Casp3
Sesn2
Atg16l1
Bcl2
Bak1
Casp8
Becn1
Ulk1
Atg12
Wipi1
Casp7
Gabarapl1
Foxo3
Sqstm1
Gabarap
Atg5
Map1lc3a
Map1lc3b
Casp9
HB8_DTT_vs_control
DUL_DTT_vs_control
HB8_vs_DUL_control
HB8_vs_DUL_DTT
1
0.5
0
-0.5
-1

Cell surface / adhesion / cytoskeleton remodeling log2FC

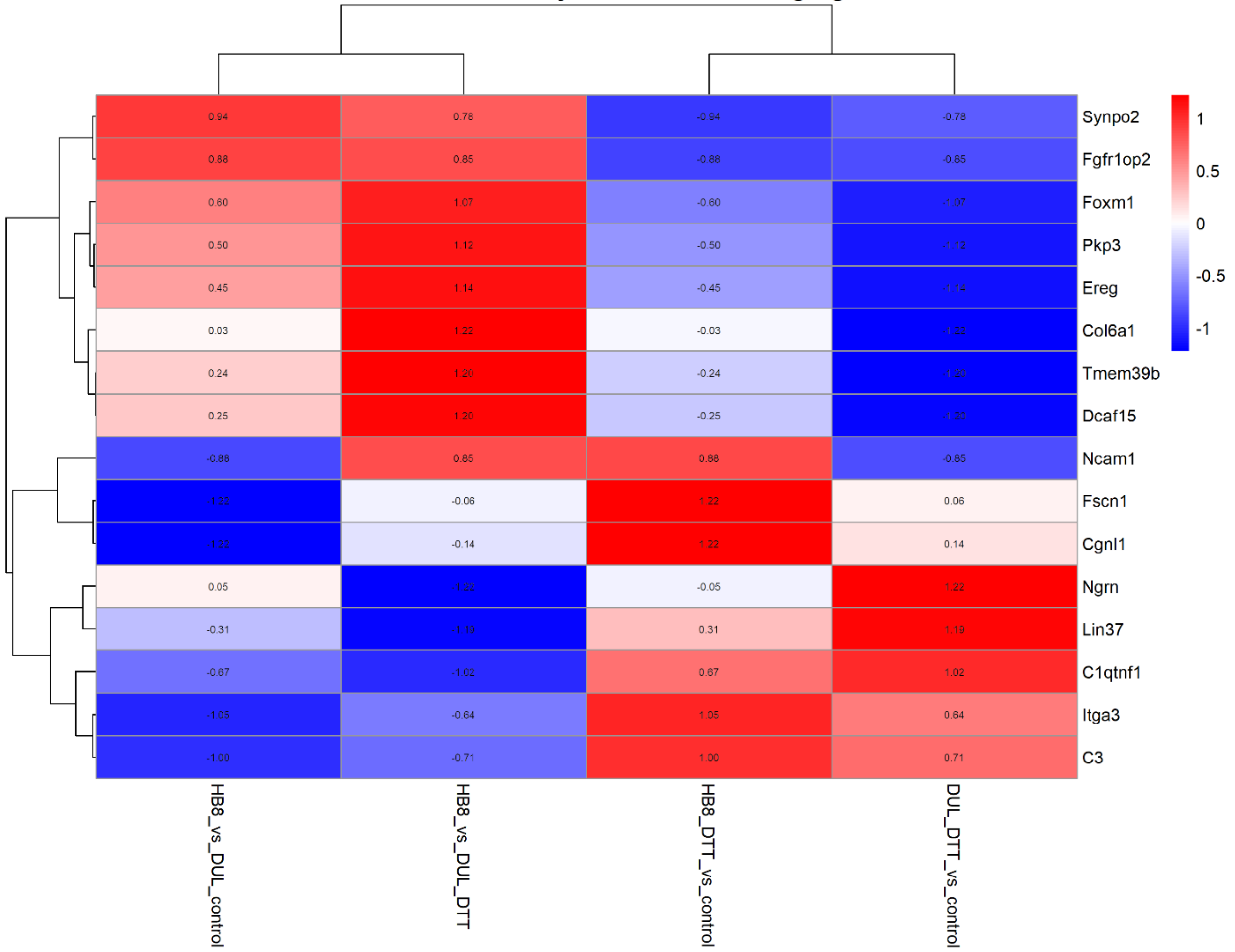

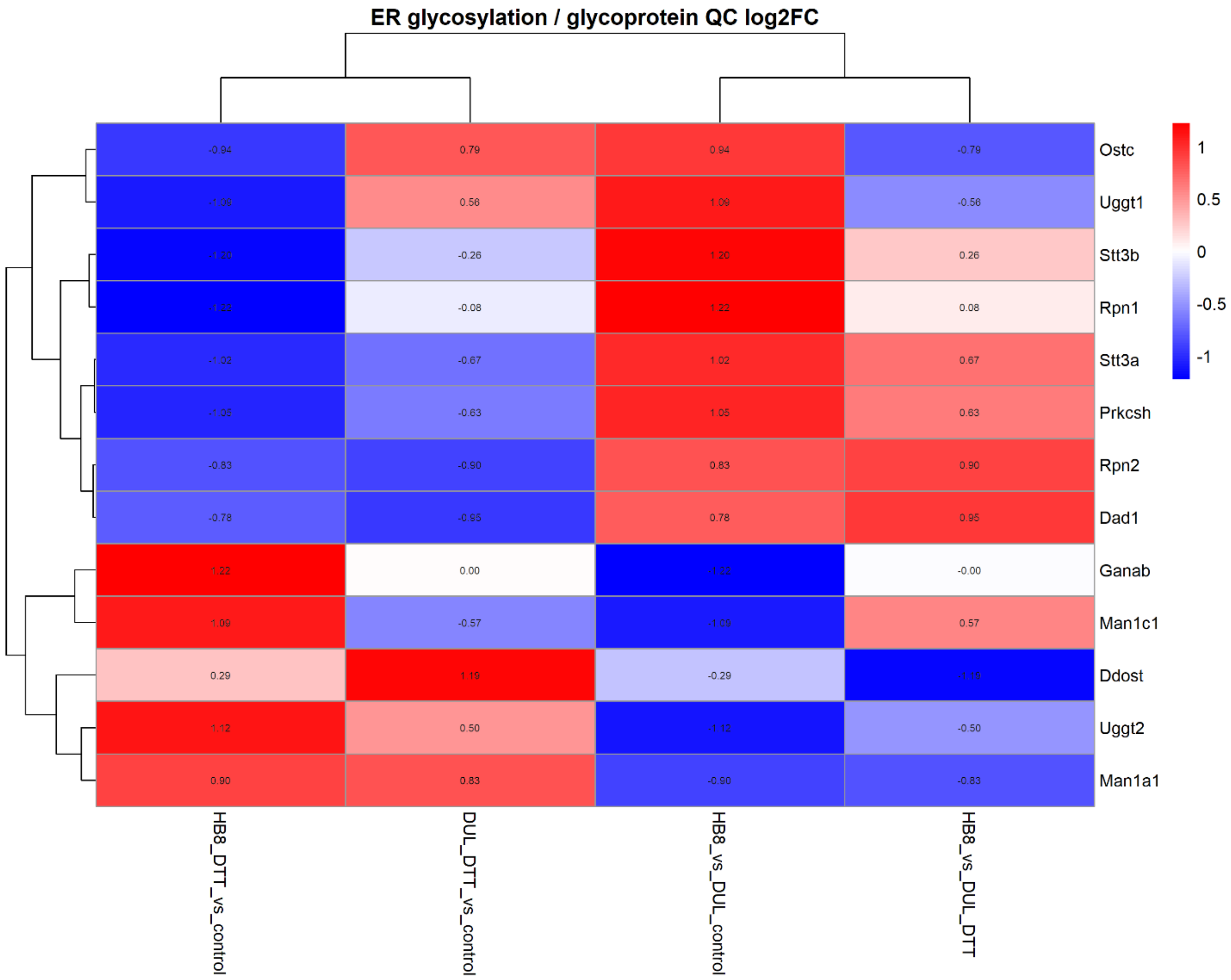
ER glycosylation / glycoprotein QC log2FC
Ostc
Uggt1
Stt3b
Rpn1
Stt3a
Prkcsh
Rpn2
Dad1
Ganab
Man1c1
Ddost
Uggt2
Man1a1
HB8_DTT_vs_control
DUL_DTT_vs_control
HB8_vs_DUL_control
HB8_vs_DUL_DTT
1
0.5
0
-0.5
-1
-0.94 0.79 0.94 -0.79
-1.09 0.56 1.09 -0.56
-1.20 -0.26 1.20 0.26
-1.22 -0.08 1.22 0.08
-1.02 -0.67 1.02 0.67
-1.05 -0.63 1.05 0.63
-0.83 -0.90 0.83 0.90
-0.78 -0.95 0.78 0.95
1.22 0.00 -1.22 -0.00
1.09 -0.57 -1.09 0.57
0.29 1.19 -0.29 -1.19
1.12 0.50 -1.12 -0.50
0.90 0.83 -0.90 -0.83

Golgi / vesicle trafficking / COPI-COPII / post-Golgi transport log2FC

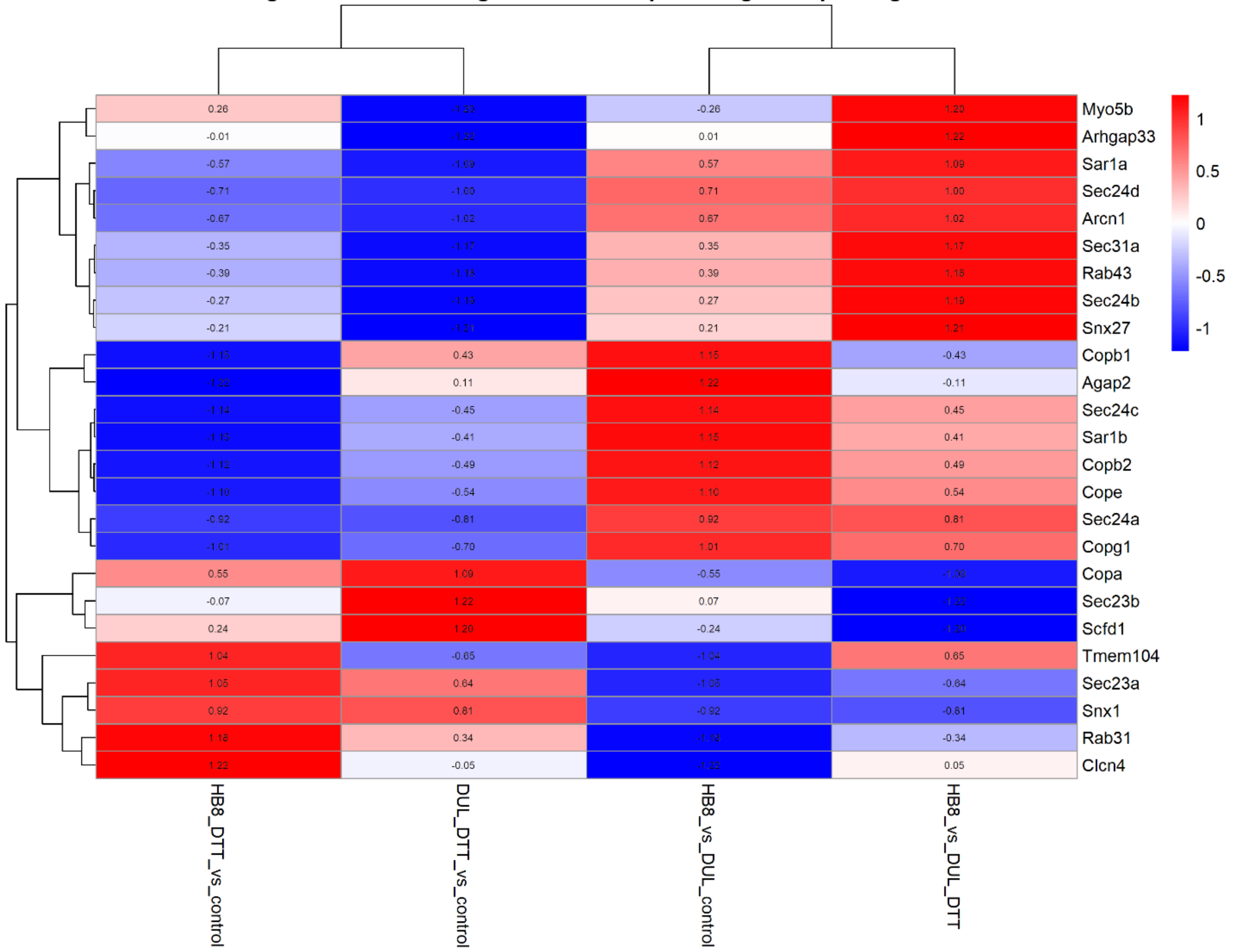

Golgi glycosylation / product-specific PTM capacity log2FC

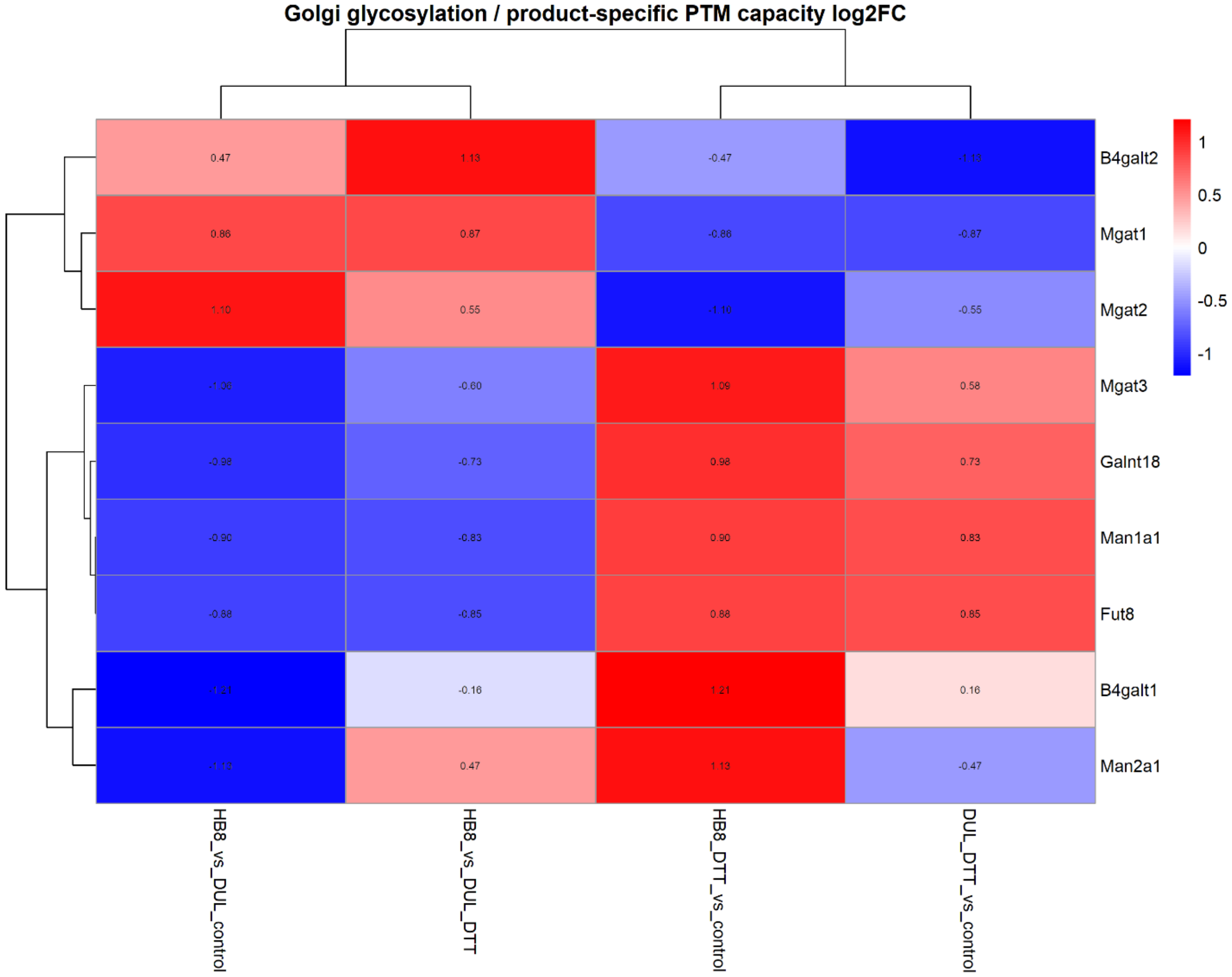

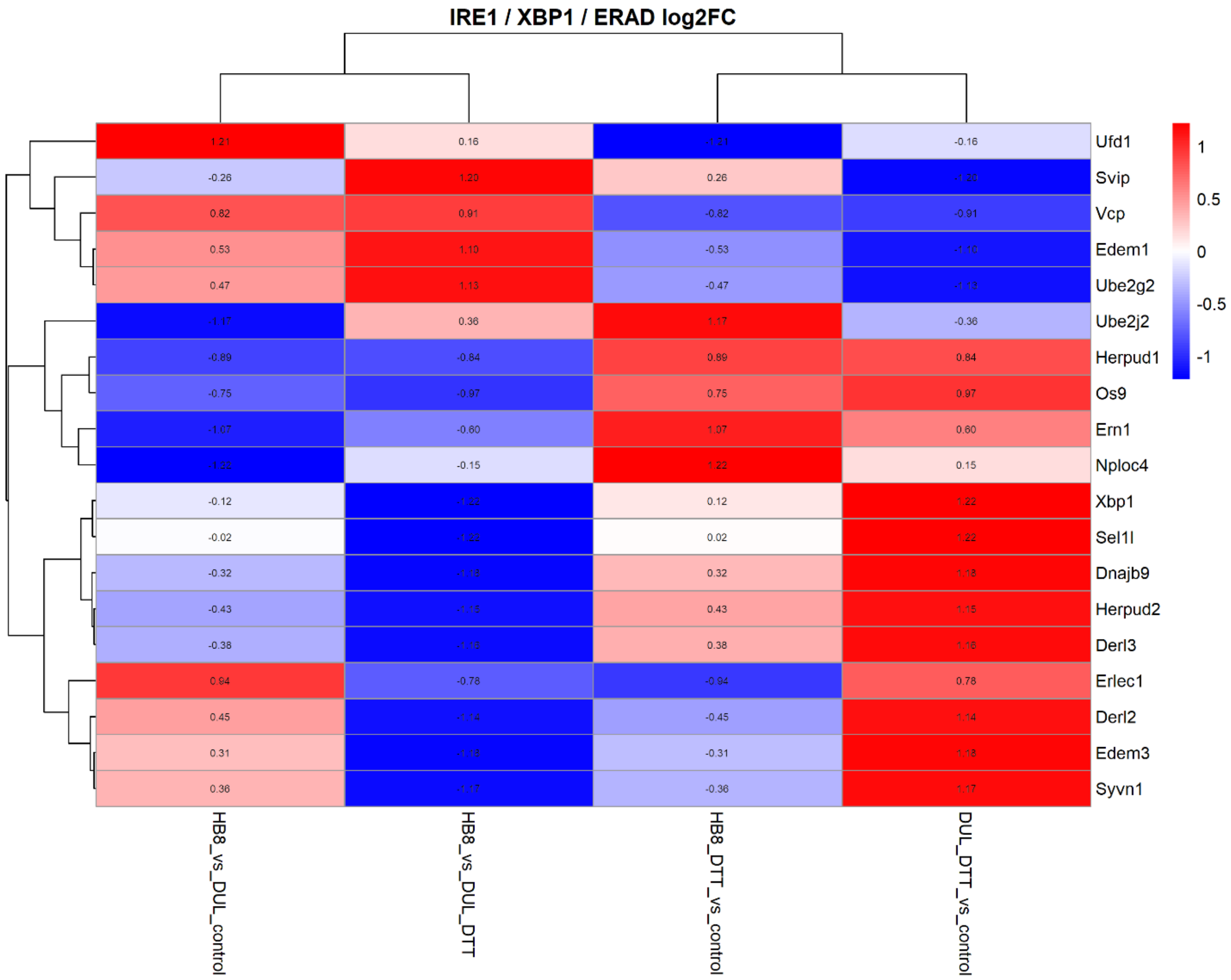
IRE1 / XBP1 / ERAD log2FC
Ufd1
Svip
Vcp
Edem1
Ube2g2
Ube2j2
Herpud1
Os9
Ern1
Nploc4
Xbp1
Sel1l
Dnajb9
Herpud2
Derl3
Erlec1
Derl2
Edem3
Syvn1
1
0.5
0
-0.5
-1
HB8_vs_DUL_control
HB8_vs_DUL_DTT
HB8_DTT_vs_control
DUL_DTT_vs_control

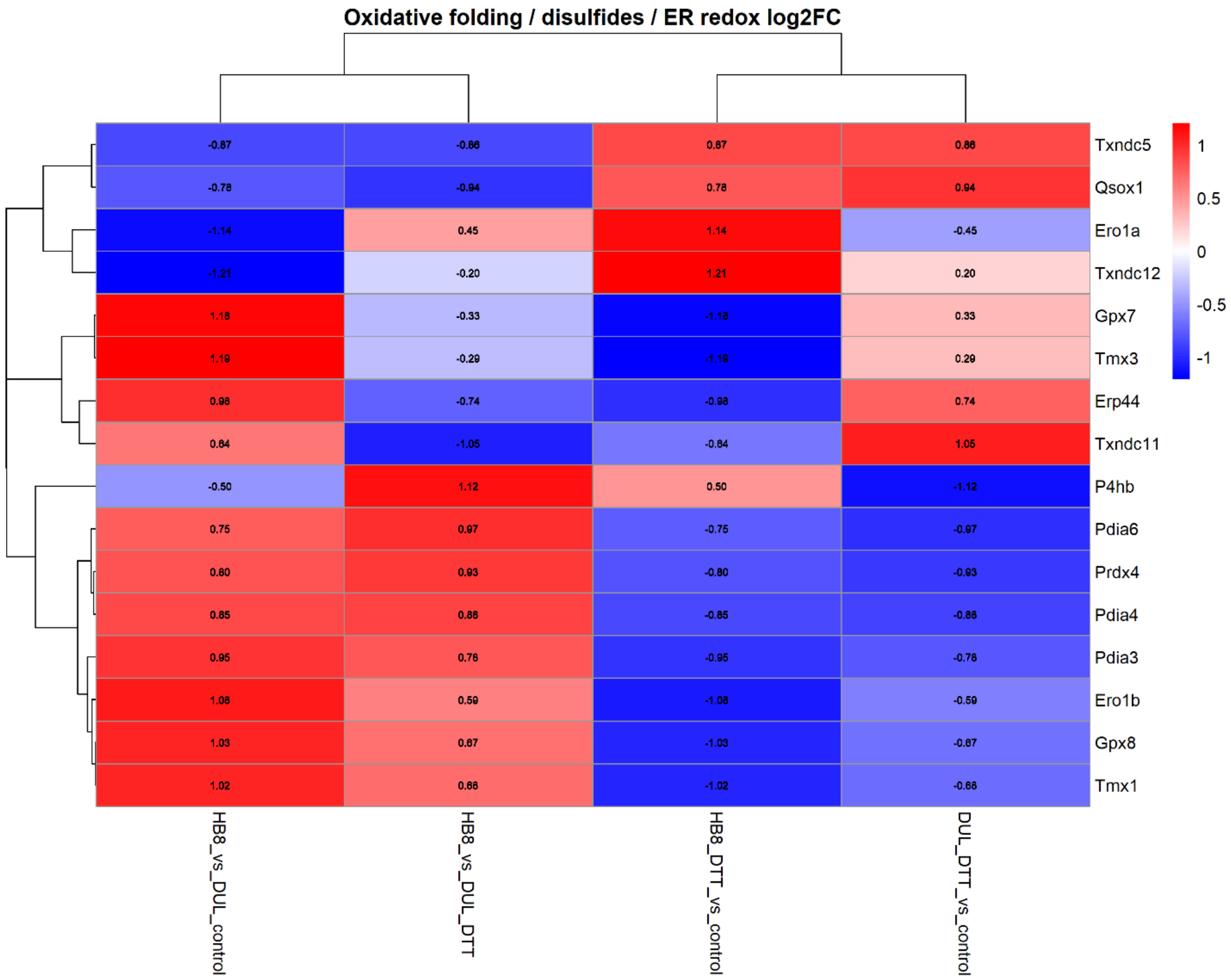
Oxidative folding / disulfides / ER redox log2FC
Txndc5: -0.87, -0.86, 0.87, 0.86
Qsox1: -0.78, -0.94, 0.78, 0.94
Ero1a: -1.14, 0.45, 1.14, -0.45
Txndc12: -1.21, -0.20, 1.21, 0.20
Gpx7: 1.18, -0.33, -1.18, 0.33
Tmx3: 1.19, -0.29, -1.19, 0.29
Erp44: 0.98, -0.74, -0.98, 0.74
Txndc11: 0.64, -1.05, -0.64, 1.05
P4hb: -0.50, 1.12, 0.50, -1.12
Pdia6: 0.75, 0.97, -0.75, -0.97
Prdx4: 0.80, 0.93, -0.80, -0.93
Pdia4: 0.85, 0.88, -0.85, -0.88
Pdia3: 0.95, 0.78, -0.95, -0.78
Ero1b: 1.08, 0.59, -1.08, -0.59
Gpx8: 1.03, 0.67, -1.03, -0.67
Tmx1: 1.02, 0.68, -1.02, -0.68
HB8_vs_DUL_control
HB8_vs_DUL_DTT
HB8_DTT_vs_control
DUL_DTT_vs_control
1
0.5
0
-0.5
-1

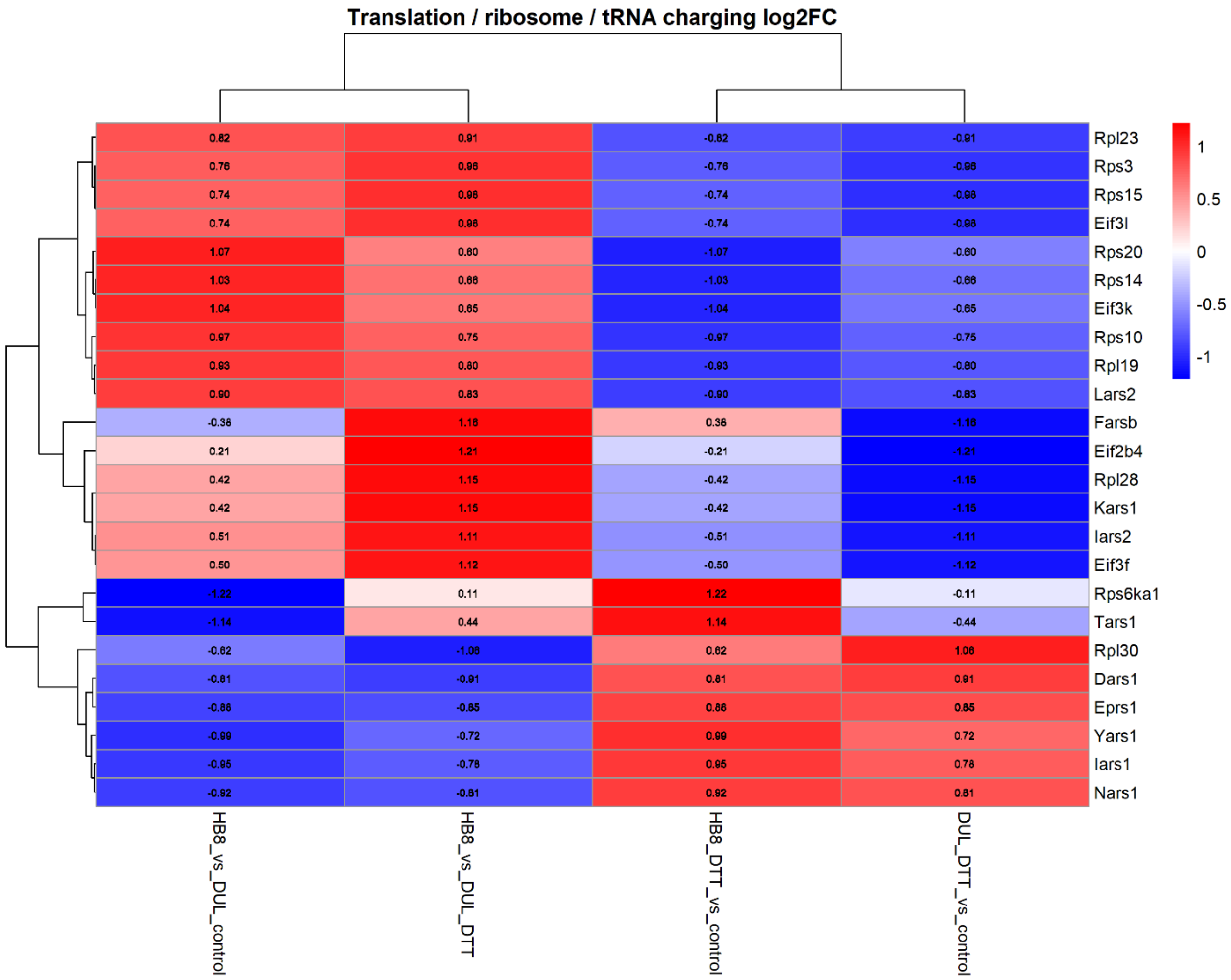
Translation / ribosome / tRNA charging log2FC
Rpl23 0.82 0.91 -0.82 -0.91
Rps3 0.76 0.96 -0.76 -0.96
Rps15 0.74 0.98 -0.74 -0.98
Eif3l 0.74 0.98 -0.74 -0.98
Rps20 1.07 0.60 -1.07 -0.60
Rps14 1.03 0.66 -1.03 -0.66
Eif3k 1.04 0.65 -1.04 -0.65
Rps10 0.97 0.75 -0.97 -0.75
Rpl19 0.93 0.80 -0.93 -0.80
Lars2 0.90 0.83 -0.90 -0.83
Farsb -0.38 1.16 0.38 -1.16
Eif2b4 0.21 1.21 -0.21 -1.21
Rpl28 0.42 1.15 -0.42 -1.15
Kars1 0.42 1.15 -0.42 -1.15
Iars2 0.51 1.11 -0.51 -1.11
Eif3f 0.50 1.12 -0.50 -1.12
Rps6ka1 -1.22 0.11 1.22 -0.11
Tars1 -1.14 0.44 1.14 -0.44
Rpl30 -0.62 -1.06 0.62 1.06
Dars1 -0.81 -0.91 0.81 0.91
Eprs1 -0.86 -0.85 0.86 0.85
Yars1 -0.99 -0.72 0.99 0.72
Iars1 -0.95 -0.78 0.95 0.78
Nars1 -0.92 -0.81 0.92 0.81
HB8_vs_DUL_control
HB8_vs_DUL_DTT
HB8_DTT_vs_control
DUL_DTT_vs_control
1
0.5
0
-0.5
-1

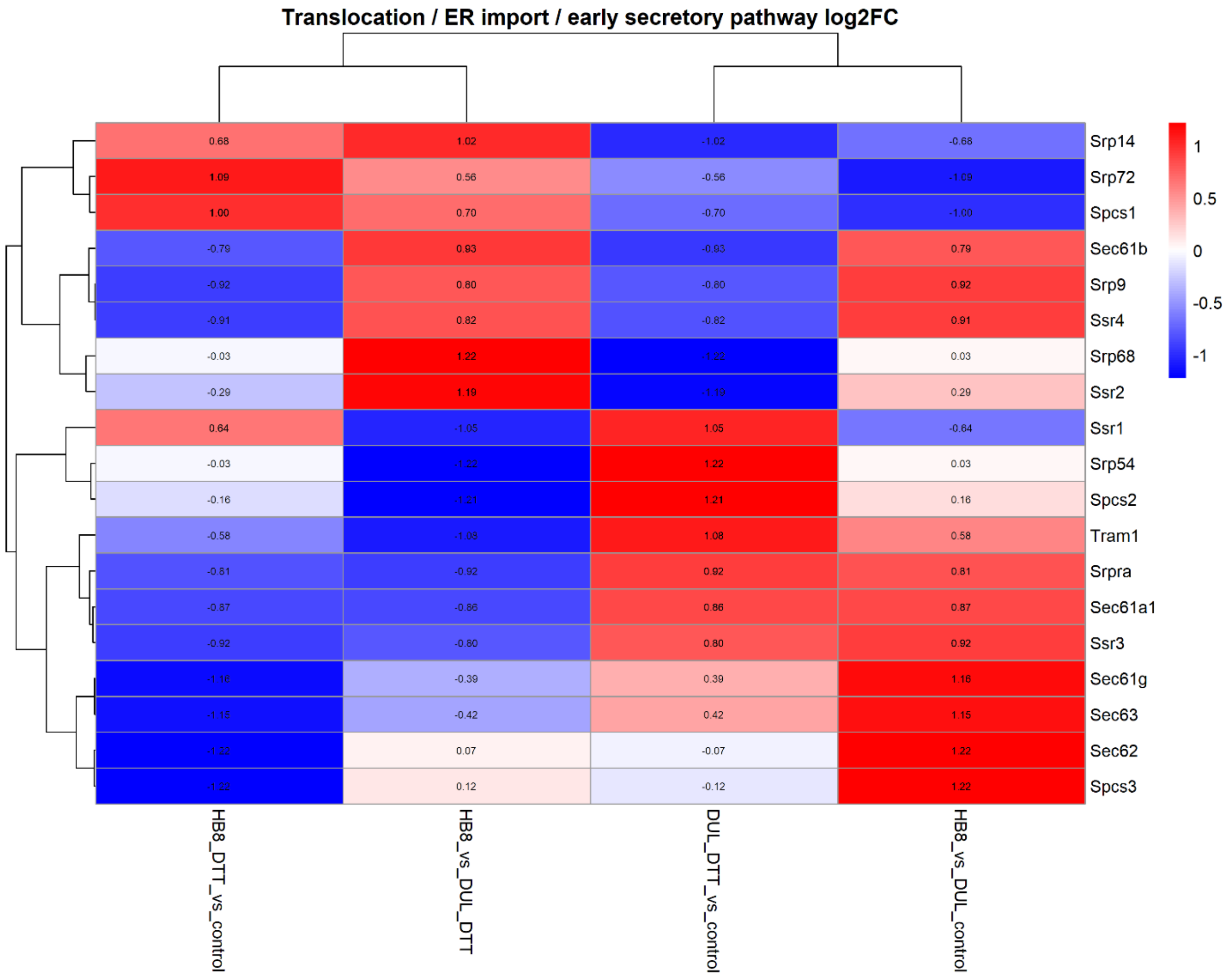
Translocation / ER import / early secretory pathway log2FC
Srp14
Srp72
Spcs1
Sec61b
Srp9
Ssr4
Srp68
Ssr2
Ssr1
Srp54
Spcs2
Tram1
Srpra
Sec61a1
Ssr3
Sec61g
Sec63
Sec62
Spcs3
HB8_DTT_vs_control
HB8_vs_DUL_DTT
DUL_DTT_vs_control
HB8_vs_DUL_control
1
0.5
0
-0.5
-1

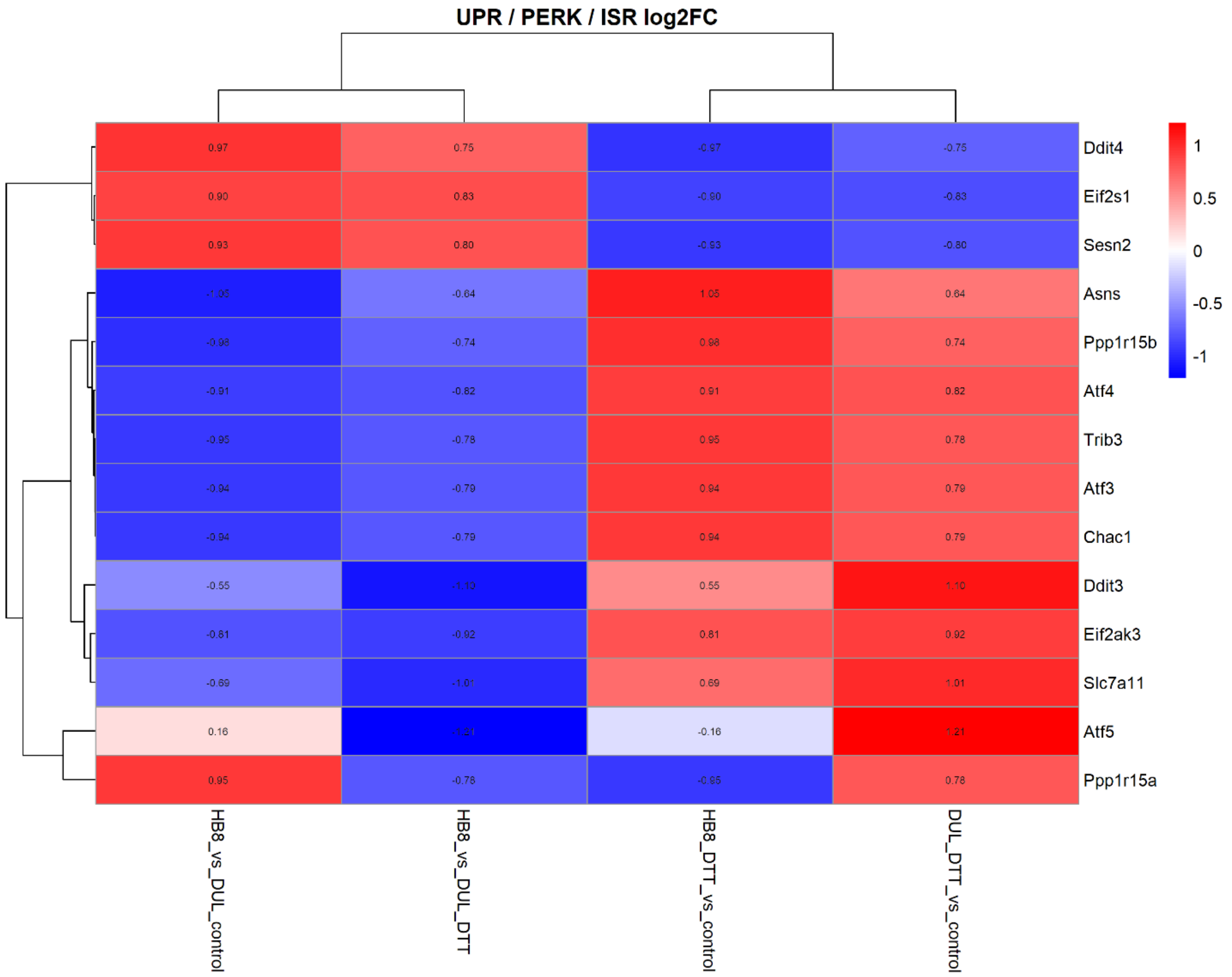
UPR / PERK / ISR log2FC
Ddit4
Eif2s1
Sesn2
Asns
Ppp1r15b
Atf4
Trib3
Atf3
Chac1
Ddit3
Eif2ak3
Slc7a11
Atf5
Ppp1r15a
HB8_vs_DUL_control
HB8_vs_DUL_DTT
HB8_DTT_vs_control
DUL_DTT_vs_control
1
0.5
0
-0.5
-1
