# Supplementary material for: DTT-Induced Transcriptome Profiling Reveals Divergent ER Proteostasis and Stress-Adaptation Programs in CHO-S/HB8 and CHO-4BGD/DUL Producer Clones
Source: Int J Mol Sci. 2026 Aug 19;27(16):7427. doi: 10.3390/ijms27167427 (PMC13513848; doi:10.3390/ijms27167427)
Supplement: Supplementary file 1 [file ijms-27-07427-s001.zip › Supplementary_Figure_S2.pdf]

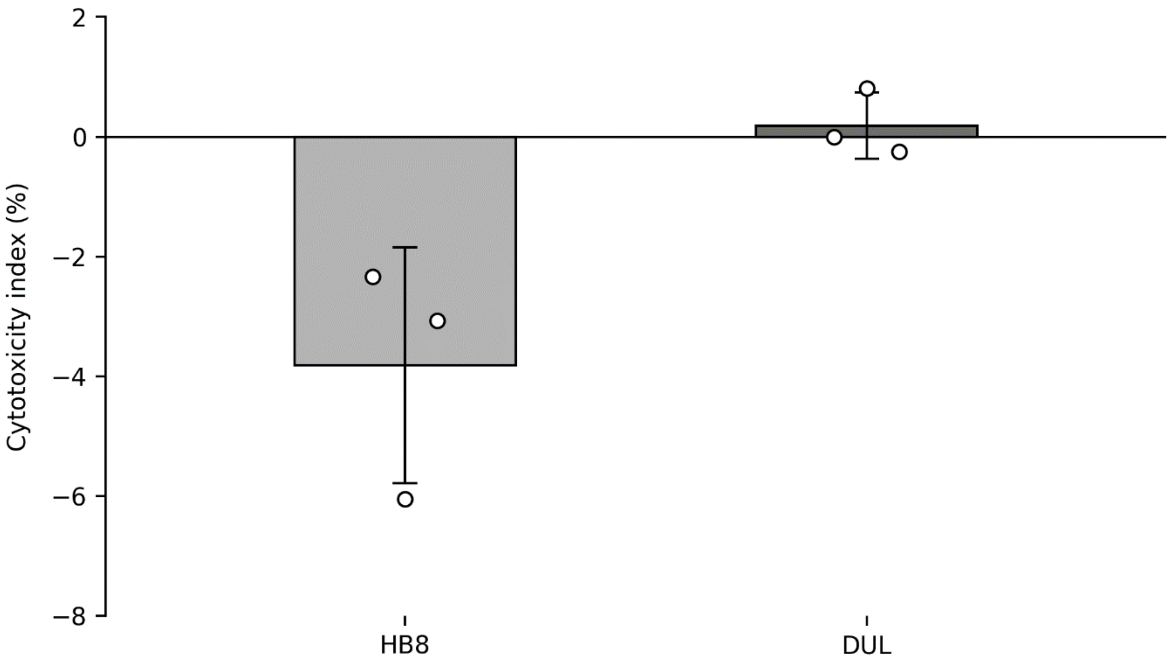

Figure S2. Cytotoxicity index in independently cultured HB8 and DUL cells after exposure to 2 mM DTT for 24 h. The cytotoxicity index was calculated relative to the corresponding untreated culture and the dilution-corrected maximum LDH activity after Triton X-100 lysis. Bars show mean ± SD of three paired biological replicates; individual values are shown as points. Negative values indicate that extracellular LDH activity after DTT treatment was lower than the spontaneous release measured in the corresponding untreated culture.
